# Hybrid nanovesicles promote diabetic wound healing via dual-targeted multimodal therapy

**DOI:** 10.1093/burnst/tkag004

**Published:** 2026-01-11

**Authors:** Zhichao Ruan, Yi Zheng, Guoyong Jiang, Jing Chen, Jiahe Guo, Chengqi Yan, Dong Liu, Shuoyuan Liu, Yufeng Wang, Pengjuan Nie, Diandian Li, Zijie Chen, Jia Tian, Zhenbing Chen, Xiaofan Yang

**Affiliations:** Department of Hand Surgery, Union Hospital, Tongji Medical College, Huazhong University of Science and Technology, Wuhan 430022, China; Department of Hand Surgery, Union Hospital, Tongji Medical College, Huazhong University of Science and Technology, Wuhan 430022, China; Department of Breast Surgery, The Second Affiliated Hospital of Nanchang University, No. 1 Minde Road, Donghu District, Nanchang 330006, Jiangxi Province, China; Department of Dermatology, Wuhan No. 1 Hospital, No. 215 Zhongshan Avenue, Jiang'an District, Wuhan 430022, Hubei Province, China; Department of Plastic Surgery and Regenerative Medicine, Fujian Medical University Union Hospital, No. 29 Xinquan Road, Gulou District, Fuzhou 350001, Fujian Province, China; Department of Hand Surgery, Union Hospital, Tongji Medical College, Huazhong University of Science and Technology, Wuhan 430022, China; Department of Radiation and Medical Oncology, Zhongnan Hospital of Wuhan University, No. 169 Donghu Road, Wuchang District, Wuhan 430071, Hubei Province, China; Department of Hand Surgery, Union Hospital, Tongji Medical College, Huazhong University of Science and Technology, Wuhan 430022, China; Department of Hand Surgery, Union Hospital, Tongji Medical College, Huazhong University of Science and Technology, Wuhan 430022, China; Department of Hand Surgery, Union Hospital, Tongji Medical College, Huazhong University of Science and Technology, Wuhan 430022, China; Department of Hand Surgery, Union Hospital, Tongji Medical College, Huazhong University of Science and Technology, Wuhan 430022, China; Department of Hand Surgery, Union Hospital, Tongji Medical College, Huazhong University of Science and Technology, Wuhan 430022, China; Department of Hand Surgery, Union Hospital, Tongji Medical College, Huazhong University of Science and Technology, Wuhan 430022, China; Department of Hand Surgery, Union Hospital, Tongji Medical College, Huazhong University of Science and Technology, Wuhan 430022, China; Hubei Provincial Clinical Research Center for Chronic Wound and Diabetic Foot, Wuhan 430077, China; Department of Hand Surgery, Union Hospital, Tongji Medical College, Huazhong University of Science and Technology, Wuhan 430022, China; Hubei Key Laboratory of Regenerative Medicine and Multi-disciplinary Translational Research (Huazhong University of Science and Technology), Wuhan 430022, China

**Keywords:** Diabetic wound, Engineering nanovesicles, Targeted delivery, Antioxidants, Macrophage polarization

## Abstract

**Background:**

Diabetic wounds remain difficult to treat due to persistent oxidative stress, chronic inflammation, and vascular dysfunction. These factors reinforce each other, forming a vicious cycle that leads to delayed healing, poor angiogenesis, and high amputation risk. Existing therapies often fail because they are unable to address these challenges simultaneously. Therefore, this study aimed to develop a hybrid extracellular vesicle system that targets these multiple barriers concurrently to promote diabetic wound healing.

**Methods:**

A biohybrid nanovesicle system (DFO@HEVs) was built by fusing endothelial cell-derived extracellular vesicles with neutrophil-derived nanovesicles (forming hybrid extracellular vesicles, HEVs), which were loaded with deferoxamine (DFO). The vesicles were tested for their physicochemical properties, drug loading, and safety. Therapeutic effects were studied *in vitro* using HG/PA-stimulated endothelial cells and macrophages and *in vivo* in diabetic mouse wounds. The analyses included microscopy, flow cytometry, histology, transcriptomics, and database-based single-cell RNA sequencing.

**Results:**

DFO@HEVs showed dual targeting: homing to endothelial cells via CXCR4 and to inflamed sites via β2 integrin. They enhanced endothelial uptake, promoted angiogenesis through PI3K/AKT/HIF-1α and VEGF signaling pathways, and reduced oxidative stress and ferroptosis by activating Nrf2 and upregulating antioxidant genes. They also shifted macrophages toward an anti-inflammatory M2 phenotype, boosted efferocytosis, and suppressed NF-κB/NLRP3-driven inflammation. In diabetic mice, treatment with DFO@HEVs accelerated wound closure, re-epithelialization, collagen deposition, and new vessel formation, while lowering neutrophil infiltration, reactive oxygen species levels, ferroptosis, and pro-inflammatory cytokines, creating a healing-supportive environment.

**Conclusions:**

DFO@HEVs provided a hybrid nanovesicle system for combined membrane and drug delivery. By promoting angiogenesis, limiting ferroptosis, and resolving inflammation, they disrupted the cycle that prevented diabetic wound repair. This approach shows a strong potential as a new treatment for chronic wounds.

## Graphical Abstract

**Graphical Abstract:**
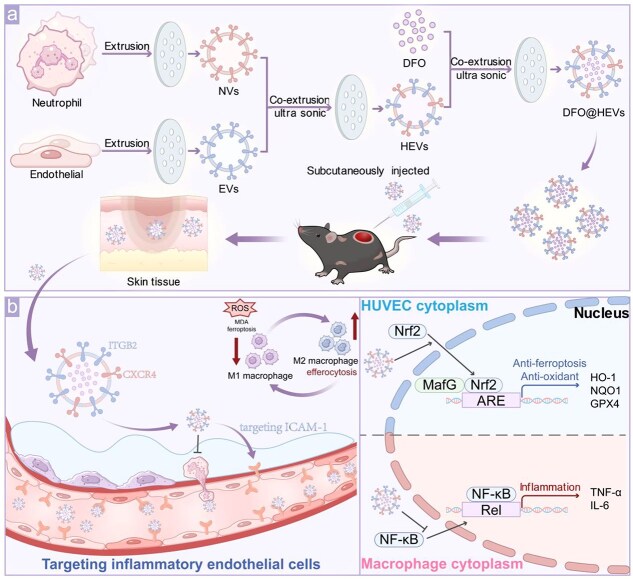

## Highlights

- **Multifunctional hybrid extracellular vesicles:** Hybrid nanovesicles derived from HUVECs and neutrophils serve as dual-targeting drug delivery vehicles with inherent anti-inflammatory and antioxidant properties.
- **Dual-targeted delivery:** DFO@HEVs achieve dual-targeted therapy by co-engaging CXCR4-mediated vascular regeneration and β2 integrin-dependent inflammation neutralization, synergistically restoring diabetic wound homeostasis.
- **Ferroptosis inhibition:** Iron chelation by DFO suppresses lipid peroxidation and restores GPX4 activity, breaking the oxidative stress-ferroptosis cycle.
- **Macrophage reprogramming:** Phosphatidylserine-enriched nanovesicles drive M2 polarization, resolving chronic inflammation and enhancing efferocytosis.

## Background

The global prevalence of diabetes is continuously increasing, and diabetic wounds are among the most devastating complications. These diabetic wounds are characterized by delayed healing, chronic inflammation, and a high risk of amputation, thereby resulting in significant clinical and economic burdens [1–3]. Current therapeutic approaches are inadequate due to the incomplete understanding of the multifactorial pathological environment underlying diabetic wounds [3, 4].

The diabetic wound microenvironment is driven by the combined effects of hyperglycemia and lipotoxicity and maintains a self-amplifying cycle of oxidative stress and chronic inflammation [5, 6]. Hyperglycemia disrupts mitochondrial redox homeostasis, leading to the excessive production of reactive oxygen species (ROS) that damages endothelial cells and amplifies inflammatory signaling. Endothelial dysfunction is characterized by the reduced bioavailability of nitric oxide (NO), impaired angiogenic capacity, and compromised vascular integrity and causes persistent tissue hypoperfusion and delayed formation of granulation tissue. Collectively, these vascular and oxidative abnormalities make a hostile wound microenvironment that significantly impairs healing in diabetic patients [6–12].

Inflammatory dysregulation is a major factor in the impairment of diabetic wound healing. Neutrophils are excessively recruited to the wound site, where they remain attached to the endothelial and tissue surfaces for a prolonged period. This leads to aggravated local damage, delayed resolution, and obstructed tissue regeneration [13]. Moreover, macrophages, which are the key regulators of inflammation and repair, exhibit dysfunctional polarization under diabetic conditions. Metabolic stress skews macrophages toward a persistent pro-inflammatory M1 state, leading to insufficient transitioning of macrophages to the anti-inflammatory M2 phenotype [14–17]. This leads to prolonged inflammation and disrupted extracellular matrix (ECM) remodeling and angiogenic signaling. Therefore, the coordinated regulation of neutrophil adhesion and macrophage polarization is essential for restoring immune balance and supporting effective wound healing.

Deferoxamine (DFO), a clinically approved iron chelator, is a promising therapeutic agent for diabetic wound healing [18–20]. It can suppress ROS generation by inhibiting the Fenton reaction and stabilize hypoxia-inducible factor-1α (HIF-1α), thus reactivating VEGF-mediated angiogenic signaling. In addition, it can also modulate iron-dependent macrophage pathways, such as the inhibition of NF-κB signaling and NLRP3 inflammasome activation, which are the key drivers of chronic oxidative inflammation [21, 22]. However, the therapeutic efficacy of DFO is limited by rapid systemic clearance, low cellular uptake, and susceptibility to degradation in the wound microenvironment, similar to the conventional drugs currently being used for treatment of diabetic wounds [23, 24]. These challenges highlight the need for advanced delivery systems, which can enable precise spatiotemporal control of drug release, increase target site accumulation, and prolong therapeutic iron chelation, which are the critical factors for effective diabetic wound healing.

Extracellular vesicles have low immunogenicity, intrinsic targeting capabilities, and excellent biocompatibility, thereby emerging as promising drug delivery carriers [25]. Compared to artificially manufactured drug delivery carriers such as liposomes, human cells-derived extracellular vesicles exhibit superior biocompatibility [26]. They also exhibit excellent stability in the body, particularly in the bloodstream, enabling stable local or systemic drug delivery [27–30]. extracellular vesicles also exhibit unique stability in harsh physicochemical environments, such as low pH or low ionic strength, and can endure rigorous processes, such as freeze-drying [31, 32]. Therefore, they extracellular vesicles can be stably stored for long periods and used in treating diabetic wounds with complex microenvironments, protecting the encapsulated drugs from degradation [33, 34]. The bioengineered nanovesicles produced through membrane extrusion retain the surface proteins and biological properties of their parental cells [35, 36]. Endothelial cell-derived extracellular vesicles (EVs) express CXCR4, which enables them homing to vascular tissues, thereby promoting angiogenesis [37–39]. In contrast, neutrophil-derived extracellular vesicles (NVs) target inflamed regions via integrin β2 (ITGB2)-ICAM-1 interactions while adsorbing inflammatory cytokines and competitively blocking neutrophil adhesion [40–43]. Nevertheless, the single-source extracellular vesicles show limited therapeutic efficacy, lacking sufficient anti-inflammatory effects, while NVs exhibit minimal angiogenic activity [44].

In order to overcome these limitations, a hybrid EV (HEV) system was developed by fusing EVs and NVs, followed by DFO encapsulation (DFO@HEVs). This dual-targeted nanoplatform combined the CXCR4-mediated endothelial homing with ITGB2-guided inflammation targeting, thus enabling precise drug delivery to diabetic wounds. By integrating angiogenic stimulation, ferroptosis inhibition, and immune modulation, DFO@HEVs offer a multifunctional strategy to remodel the diabetic wound microenvironment and promote effective healing.

## Methods

### Reagents

Palmitic acid (PA), DFO, protease inhibitor cocktail, and glucose were procured from MCE (Shanghai, China). Lipopolysaccharide (LPS, *Escherichia coli* O111:B4) was obtained from Sigma–Aldrich (USA). The membrane protein extraction kits, penicillin–streptomycin solution, fetal bovine serum (FBS), Hank’s balanced salt solution (HBSS), and Dulbecco’s modified Eagle’s medium (DMEM) were supplied by Gibco (USA). DiR, 4′,6-diamidino-2-phenylindole (DAPI), 2′,7′-dichlorofluorescein diacetate (DCFH-DA), the Griess reagent kit, the BCA assay kit, DiO, DiI, DiD, and Actin-Tracker Green-488 were purchased from Beyotime (China). Mouse IL-6, IL-10, and TNF-α ELISA kits were acquired from ELK Biotechnology (Wuhan, China). Other chemical reagents and solvents were obtained from Sinopharm Chemical Reagent Co., Ltd (China). Antibodies for western blot, immunofluorescence, and flow cytometry detection were obtained from Abcam, Cell Signaling Technology, BioLegend, Santa Cruz Biotechnology, Invitrogen (USA), Abmart, and Proteintech (China).

### Animals

Male C57BL/6 mice (8 to 10 weeks old) were obtained from the Hubei Provincial Center for Disease Prevention and Control (Wuhan, China). All specific-pathogen-free (SPF) mice were housed in the Animal Center of Huazhong University of Science and Technology under controlled pathogen-free conditions. Animal experiments were conducted according to the principles approved by the Institutional Animal Care and Use Committee (IACUC) of Tongji Medical College, Huazhong University of Science and Technology (protocol number: 4452 [2024]).

### Cell lines and culture

Human umbilical vein endothelial cells (HUVECs, #GDC166), HaCaT cells (#GDC106), and 293T cells (#GDC0187) were purchased from the China Center of Type Culture Collection (CCTCC, Wuhan, China). Immortalized murine bone marrow macrophages (iBMDMs, #M3–1001) were obtained from OriCell (Guangzhou, China). Human HL-60 cells (#CL-0110) and human Jurkat clone E6 cells (#CL-0129) were purchased from Procell Life Science & Technology (Wuhan, China). All the cell lines were cultured according to the supplier’s instructions. Neutrophils were differentiated from HL-60 cells through a 5-day induction protocol with 1 μM all-trans retinoic acid and 1.25% dimethyl sulfoxide. The percentage of CD11b^+^/CD66b^+^ cells in HL-60 cells was determined using flow cytometry to assess the neutrophil induction effect (Figure S1a, see online supplementary material).

### Manufacture and isolation of DFO@HEVs

Extracellular vesicles derived from endothelial cells were prepared following previously described methods with modifications. Briefly, cultured HUVECs were dissociated via TrypLE™ Express Enzyme (Gibco, USA) and diluted to 5 × 10^6^ cells/ml in PBS. The cell suspension was extruded through a series of Nuclepore™ polycarbonate membranes (10 μm, 5 μm, 1 μm, 0.4 μm, and 0.2 μm pore sizes) via a miniextruder (Avanti Polar Lipids, USA) under ambient temperature and pressure. Each extrusion step was repeated 10 times. Neutrophil-derived extracellular vesicles (NVs) were manufactured similarly.

Hybrid extracellular vesicles (HEVs) were produced by first preparing EVs and NVs individually via membrane extrusion. The two types of vesicles were then mixed at a 1:1 (w/w) ratio, followed by vortexing and sonication (30% amplitude; 30 s on, 2 min off, 4–6 cycles in an ice–water bath). The mixture was subsequently extruded 10 times through a 0.2 μm polycarbonate membrane using an Avanti mini-extruder and incubated at 37°C for 1 hour to facilitate membrane fusion.

For DFO loading, HEVs were mixed with DFO at a 1:2 (w/w) ratio and subjected to ultrasonication at 30% amplitude using 10-s pulses followed by 10-s intervals for 4–6 cycles in an ice–water bath. The resulting mixture was then extruded 10 times through a 0.2 μm polycarbonate membrane using an Avanti mini-extruder and incubated at 37°C for 2 hours to obtain DFO@HEVs.

The vesicles were isolated via differential centrifugation. The extruded vesicle suspension was first centrifuged at 3000 × g for 45 minutes at 4°C to remove large debris, followed by centrifugation at 10 000 × g for 45 minutes at 4°C to pellet microvesicles. The resulting supernatant was then subjected to ultracentrifugation at 140000 × g for 70 minutes at 4°C to collect nanoscale vesicles. The pelleted vesicles were washed twice with sterile PBS and finally resuspended in PBS for storage at −80°C until use.

### Characterization of DFO@HEVs

Transmission electron microscopy (TEM) was used to visualize the structure of extracellular vesicles. Ten microliters of isolated extracellular vesicles were placed on copper grids for 5–10 minutes, and excess liquid was blotted with filter paper. After air-drying, the samples were stained with 2% uranyl acetate (10 μl) and imaged via an HT7700 transmission electron microscope (Hitachi, Japan). Nanoparticle tracking analysis (NTA) was performed to measure the particle size and concentration via a Zeta View PMX-120 system (Particle Metrix, Germany).

### Verification of HEV fusion

To confirm EV-NV fusion, DiI-labeled EVs (DiI@EVs) and DiO-labeled NVs (DiO@NVs) were used to generate co-labeled HEVs during hydration. The colocalization of DiI and DiO fluorescence was analyzed via confocal laser scanning microscopy (CLSM) and the Förster resonance energy transfer technique.

### Residual membrane protein analysis

The membrane protein content of EVs, NVs, and HEVs was analyzed via SDS–PAGE. Proteins were denatured, separated via 10% SDS–PAGE, and stained with Coomassie blue. Specific membrane protein markers (e.g. LFA-1, CD11b, and CXCR-4) were detected via western blotting via the appropriate antibodies and enhanced chemiluminescence (ECL) kits.

### Encapsulation efficiency and release kinetics of DFO in DFO@HEVs

The concentration of DFO was evaluated via methods described by Li *et al.* [45] Briefly, 100 μl of the collected solution was reacted with the same volume of 3 mM FeCl3 solution for 10 min. According to the standard curve, the concentration of DFO in the supernatant was calculated. The EE DFO@HEVs were calculated according to the following equations.

EE (%) = (total amount of added drug-the amount of remaining drug)/(total amount of added drug) × 100%.

In short, after mixing 200 μM DFO with HEVs, drug loading was performed using either ultrasonication or extrusion. The mixture was subsequently incubated at 37°C for 2 hours to facilitate drug encapsulation. The resulting DFO@HEVs solution was then transferred to a 500 μl ultrafiltration tube (Millipore) and centrifuged at 2500 × g for 30 minutes at 4°C. The solution collected in the outer chamber of the ultrafiltration tube represented the unencapsulated free DFO, whose concentration was measured and used to calculate the encapsulation efficiency according to the formula above.

### Co-incubation method

The drug and vesicles were directly mixed and incubated together at 37°C for 2 hours. During this time, the drug naturally interacted with the vesicles and was gradually encapsulated within them.

### Ultrasonication method

The drug solution and vesicles were mixed and subjected to ultrasound at 30% power. The mixture was kept on ice and sonicated for 10 s, followed by 10 s of rest. This cycle was repeated 4–6 times. The ultrasonic waves generate local high temperatures and pressure, which help facilitate the loading of the drug into the vesicles. After ultrasonication, the mixture was incubated at 37°C in a water bath for 2 hours to promote further drug encapsulation.

### Extrusion method

After ultrasonication, the mixture of drug and vesicles was processed using a liposome extruder through a 200 nm filter. The mixture was extruded 10 times to ensure uniform encapsulation of the drug within the vesicles. After extrusion, the mixture was incubated at 37°C for 60 minutes in a water bath.

Afterward, the solution was subjected to ultrafiltration using a 100 kDa ultrafiltration membrane. The mixture was centrifuged at 5000 g at 4°C for 25 minutes. Finally, the filtrate in the bottom chamber of the ultrafiltration device was collected, and the DFO concentration was measured using the method described above to determine the drug encapsulation efficiency.

The drug release curve was measured as follows: First, 200 μg of DFO@HEVs in 5 ml of PBS was incubated at 37°C without agitation, and the supernatant was collected at each predetermined time point (6 h, 12 h, 24 h, 36 h, and 72 h). The DFO in the supernatant was subsequently chelated with iron (Fe^3+^), and a UV–vis spectrophotometer was used to measure the release concentration of DFO.

### Analysis of endothelial-targeted effects *in vitro*

Extracellular vesicles derived from endothelial cells and 293T cells were co-extruded with NVs to generate EC-HEVs and 293T-HEVs, respectively. Endothelial cells were then co-incubated with CFSE-GREEN for 10 minutes to obtain green fluorescent-labeled endothelial cells. Similarly, human dermal fibroblasts (HDFs) or keratinocytes (HaCAT cells) were stained with Hoechst 33423 via a 10-minute co-incubation to obtain DAPI-labeled HDF/HaCAT cells. After three washes with PBS, equal numbers of endothelial cells and either HDFs or HaCAT cells were co-seeded into 24-well plates. After 24 hours, the extracellular vesicles were pretreated with anti-CXCR4 or IgG antibodies for 2 hours according to the experimental grouping, followed by the addition of the differently treated vesicles. After an additional 12 hours, the cells were either imaged via confocal laser scanning microscopy or collected for flow cytometry analysis.

For confocal laser scanning microscopy, CFSE-labeled endothelial cells were co-cultured with HaCAT or fibroblasts, followed by co-staining the endothelial cells and keratinocytes or fibroblasts with DAPI. DAPI^+^/FITC^+^/PE^+^ cells were considered endothelial cell uptake, while DAPI^+^/PE^+^ cells were considered keratinocyte or fibroblast uptake.

For flow cytometry analysis, endothelial cells were co-cultured with HaCAT or HDF cells, which were pre-labeled with Hoechst. The amount of DAPI^+^/PE^+^ (keratinocyte or fibroblast uptake) and DAPI^−^/PE^+^ (endothelial cell uptake) was measured to assess the uptake efficiency.

### Analysis of endothelial targeting *in vivo*

The Dio-labeled EC-HEVs (100 μl, 20 μg/ml) and Dio-labeled 293T-HEVs (100 μl, 20 μg/ml) were injected into the wounds of the EC-HEVs and 293T-HEVs, respectively, in diabetic mice. The wound tissue collected on Day 3 was frozen and made into longitudinal sections. These sections were subsequently stained with DAPI and an anti-CD31 antibody. Fluorescence microscopy images were acquired at the wound center and edge (Nikon, Japan).

### Cellular uptake

Vesicles labeled with DiI were incubated with HUVECs for 24 hours. The cells were stained with phalloidin and DAPI to visualize the cytoskeleton and nuclei. Images were captured with a Nikon confocal microscope. The co-localization of DiI and DiO in fusion vesicles was analyzed via ImageJ.

### Analysis of inflammation-targeted effects *in vitro*

Endothelial cells were seeded into 24-well plates and cultured for 24 hours, followed by treatment with either TNF-α (20 ng/ml) or PBS for 12 hours. Subsequent pretreatments of the cells or extracellular vesicles were performed according to specific experimental requirements. Briefly, endothelial cells in different groups were pretreated with either an anti-ICAM1 antibody or an isotype control IgG for 2 hours, followed by incubation with various extracellular vesicles for 12 hours. The cells were then either imaged via confocal microscopy or collected for flow cytometry analysis.

To investigate the role of ITGB2, extracellular vesicles were first pretreated with either an anti-ITGB2 antibody or an isotype control IgG prior to co-incubation with endothelial cells. Unbound antibodies were removed via ultracentrifugation, after which the vesicles were incubated with endothelial cells according to group assignments for 12 hours before subsequent analysis.

### Cellular proliferation and migration

A high-glucose and high-palmitic acid (HG + PA) model was established by culturing HUVECs in medium supplemented with 300 μM PA and 35 mM glucose. Vesicle treatments (20 μg/ml) were applied for 12 hours prior to medium replacement. Cellular proliferation was assessed via an EdU kit and colony formation assays, whereas migration was evaluated via transwell chambers and scratch assays. Oxidative stress and hypoxia Model HUVECs were treated with 300 μM PA and 35 mM glucose to simulate oxidative stress and hypoxia. Cell viability was measured via CCK-8 and calcein-AM/PI double staining. Fluorescence microscopy was employed to capture images of the stained cells.

### Single-cell RNA sequencing analysis

Publicly available single-cell RNA sequencing data (GSE165816) were downloaded from the GEO database. The dataset includes foot skin samples from healthy individuals and patients with diabetic foot ulcers (DFUs). The raw count matrices were processed via the Seurat R package (v4.3). Quality control filters excluded cells with <200 or > 6000 detected genes and > 15% mitochondrial gene content. The data were normalized and log-transformed via the normalizeData function, followed by the identification of highly variable genes. Principal component analysis (PCA) was performed, and the top 20 principal components were used for clustering (resolution = 0.5) and UMAP dimensionality reduction. Major cell types were annotated on the basis of canonical markers. Differentially expressed genes (DEGs) between DFU patients and healthy controls were identified via the FindMarkers function (Wilcoxon test, adjusted *P* < 0.05).

For pathway analysis, DEGs from endothelial cells and macrophages were subjected to Gene Ontology (GO) and Kyoto Encyclopedia of Genes and Genomes (KEGG) enrichment analyses via the clusterProfiler package. Subcluster-specific enrichment was conducted on the basis of unsupervised re-clustering of endothelial and macrophage subsets. Functional terms with adjusted *p* values <0.05 were considered significantly enriched.

### mRNA sequencing analysis process

Sequencing and Differentially Expressed Genes Analysis The libraries were sequenced on an Illumina Novaseq 6000 platform and 150 bp paired-end reads were generated. Raw reads of fastq format were firstly processed using fastp1 and the low quality reads were removed to obtain the clean reads. Then about 47 million clean reads for each sample were retained for subsequent analyses. The clean reads were mapped to the reference genome using HISAT22. FPKM3 of each gene was calculated and the read counts of each gene were obtained by HTSeq-count4. PCA analysis were performed using R (v 3.2.0) to evaluate the biological duplication of samples. Differential expression analysis was performed using the DESeq25. Q value <0.05 and foldchange >2 or foldchange <0.5 was set as the threshold for significantly differential expression gene (DEGs). Hierarchical cluster analysis of DEGs was performed using R (v 3.2.0) to demonstrate the expression pattern of genes in different groups and samples. The radar map of top 30 genes was drew to show the expression of up-regulated or down-regulated DEGs using R packet ggradar. Based on the hypergeometric distribution, GO6, KEGG7 pathway, Reactome and WikiPathways enrichment analysis of DEGs were performed to screen the significant enriched term using R (v 3.2.0), respectively. R (v 3.2.0) was used to draw the column diagram, the chord diagram and bubble diagram of the significant enrichment term.

Gene Set Enrichment Analysis (GSEA) was performed using GSEA software8–9. The analysis was used a predefined gene set, and the genes were ranked according to the degree of differential expression in the two types of samples. Then it is tested whether the predefined gene set was enriched at the top or bottom of the ranking list.

### Ferroptosis and oxidative stress measurements

The intracellular Fe^2+^ levels were determined via ferro-orange, lipid peroxidation was assessed via BODIPY C11, and the ROS levels were measured via DCFH-DA. MDA levels and the activities of SOD and GPx were analyzed via the appropriate assay kits.

### Apoptosis assay

Fibroblast apoptosis was analyzed via an Annexin V-FITC Apoptosis Detection Kit. The stained cells were evaluated via flow cytometry and analyzed via FlowJo software.

### Anti-inflammatory effect analysis *in vitro*

To induce a proinflammatory phenotype (M1), iBMDMs were incubated for 24 hours at 37°C with 100 ng/ml LPS. To remove the inducer and other components, the pro-inflammatory BMDMs were washed three times with PBS. Subsequently, EVs, NVs, HEVs, and DFO@HEVs dispersed in blank media were added. After incubation for 24 h, the culture supernatant was collected and centrifuged at 10 000 rpm for 10 min at 4°C. The isolated supernatant was replaced in a low-adsorption centrifuge tube, and then the nitrite and cytokine levels were detected. A Griess reagent kit was used to detect the NO content. Following the manufacturer’s instructions, the level of NO secretion was detected by measuring the absorbance at 540 nm. Moreover, cytokine (IL-6, TNF-α, and IL-10) tests were performed via ELISA kits. To detect the polarization of the iBMDM phenotype, the cells were collected and incubated with Fc blocking reagent, an anti-CD16/32 antibody, and then with FITC-conjugated anti-CD86 for 30 min at 4°C. Next, the cells were fixed and permeabilized prior to being labeled with APC-conjugated anti-CD206. Pro-inflammatory iBMDMs that were not subjected to any treatment served as controls.

### *In vitro* efferocytosis assays

Jurkat T cells were labeled with CFSE dye and subjected to 30 W ultraviolet irradiation for 2 hours to induce apoptosis. A cell apoptosis kit was used to detect the degree of apoptosis via FCM. iBMDMs were seeded into confocal dishes (5 × 10^4^ cells per well) and cultured for 24 hours under inflammatory stimulation (100 ng/ml LPS). The media was subsequently replaced with different drug groups and incubated for 4 hours. Then, the culture supernatant was discarded, and CFSE-labeled apoptotic Jurkat T cells were added to the macrophages at a 4:1 ratio (apoptotic cell-to-phagocyte number ratio) in the presence of media and incubated at 37°C for 1 hour. Macrophages were washed with cold PBS to remove the suspended cells and analyzed by FCS and/or confocal imaging. The iBMDMs were labeled with Celltracker Red, and the nuclei were labeled with DAPI for confocal imaging (Nikon AX, Japan). For FCM, apoptotic Jurkat T cells were labeled with 2 μM CFSE, and efferocytosis activity was evaluated as the percentage of Celltracker Red-labeled macrophages that phagocytosed CFSE-labeled apoptotic cells.

### SDS-PAGE and western blot

The total protein of the vesicles was extracted with RIPA lysis buffer supplemented with proteinase inhibitor (Roche, Switzerland). The protein concentration was determined with a BCA protein assay kit (Beyotime, Shanghai, China). Equal amounts of total protein (20 μg) were separated via SDS–PAGE (Beyotime, China). Coomassie blue staining (Hycezmbio, China) was used to detect the protein in the harvested polyacrylamide gels.

For western blotting, the total protein was transferred to polyvinylidene fluoride membranes (Millipore, USA) after being separated by SDS–PAGE. The blocked membranes were then incubated with primary antibodies against ITGB2, CD11b， CXCR4, Nrf2, HO1, NQO1,GPX4,ACSL4, iNOS, ARG1, IKKα/β, phosphorylated IKKα/β,IKBα, phosphorylated IKBα, P65, phosphorylated P65, and NLRP3 overnight at 4°C. The membranes were subsequently incubated with secondary antibodies for 2 hours and exposed to X-ray film (UVP, CA, USA).

### Flow cytometry

The attached adipose tissue was removed before the skin wound tissue was harvested, and the skin samples were sliced and digested with Opti-MEM (Invitrogen) solutions containing collagenase type I (0.5 mg/ml, BioFroxx, China), collagenase type II (0.5 mg/ml, BioFroxx, China), collagenase type IV (1 mg/ml, BioFroxx, China), hyaluronidase (1 mg/ml, BioFroxx, China), and deoxyribonuclease I (0.02 mg/ml, Biosharp, China) for 30 min on a shaker at 37°C. The tissue was mechanically ground via a tissue grinder and washed with complete medium containing 10% FBS, after which the red blood cells were lysed.

The cell surfaces were stained in the dark on ice for flow cytometry analysis. The cells were stained with antibodies against the surface antigens CD11b (elabscience, China), Ly6G (elabscience, China) and CD86 (Thermo Fisher). The cells were fixed and permeabilized and then incubated with anti-CD16/32 (BD bioscience) to block nonspecific binding. The staining of intracellular markers, such as CD206 (Thermo Fisher), was performed on ice. The cells were then analyzed by flow cytometry.

### Diabetic wound model

All animal experiments were approved by the Animal Care Committee of Tongji Medical College. Eight-week-old male C57/BL6 mice were intraperitoneally injected with streptozotocin (STZ, 50 mg/kg) for 5 consecutive days. Blood glucose levels were measured using a glucometer at 1 week and again at 2 weeks after injection. Mice with sustained blood glucose levels above 16.7 mM were confirmed as successfully induced diabetic models (Table S1, see online supplementary material). After maintaining the diabetic condition for 4 weeks, full-thickness cutaneous wounds were created on the dorsal skin. Before surgery, a total of 30 diabetic mice were anesthetized with pentobarbital sodium (Sigma-Aldrich) (1%, 50 mg/kg). After shaving and sterilization, a full-thickness excision wound with a diameter of 10 mm was created on the back of each mouse. The wounds were treated with PBS, EVs, NVs, HEVs, or DFO@HEVs. Each wound received a subcutaneous injection of 50 μg of the respective vesicle formulation around the wound site. Wound healing was continuously monitored and analyzed using ImageJ software.

### Histological and immunofluorescence analyses

Wound tissues were fixed, embedded in paraffin, and sectioned. Granulation tissue and collagen deposition were evaluated via hematoxylin and eosin (H&E) and Masson staining, respectively. Immunofluorescence staining for CD31, α-SMA, GPX4, and TNF-α was performed, and the data were analyzed via ImageJ.

### Statistics

The statistical analyses were performed with GraphPad Prism software (version 8.4.3; La Jolla, CA, USA). Unpaired Student’s t tests were used for comparisons between two groups. One-way analysis of variance with Tukey’s post hoc test was employed for comparisons among groups ≥3. All the data are presented as the mean ± standard deviation (SD). Statistical significance was set at *P* < 0.05.

## Results

### Manufacturing and characterization of DFO@HEVs

**Figure 1:**
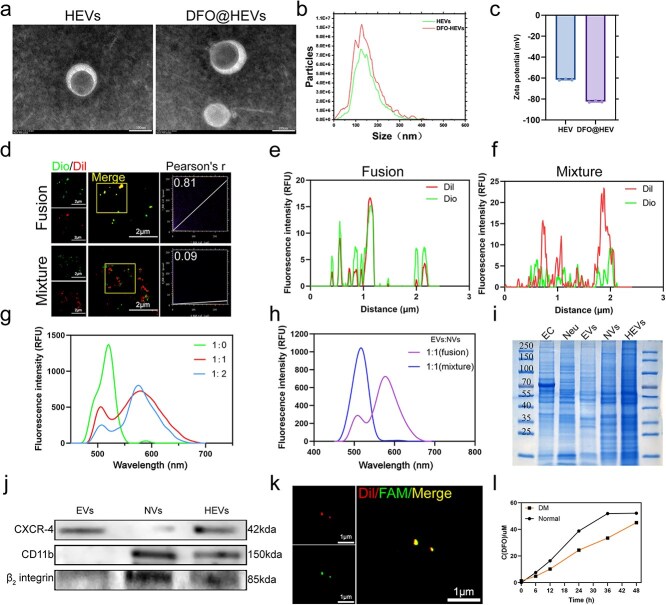
Manufacture and characterization of DFO@HEVs. (**a**) TEM image of HEVs. Scale bar: 100 nm. (**bc**) The results of particle size and zeta potential of vesicles in PBS (n = 3). (**d**) CLSM of fluorescence-labeled HEVs (red, DiI@EVs; green, DiO@NVs). Scale bar: 2 μm. (**ef**) Colocalization analysis of the fusion and physical mixture groups (n = 3). The fluorescence intensity was evaluated in the yellow field in (d). (**gh**) Verification of HEV fusion via the FRET effect. (**i**) SDS–PAGE of HUVECs, neutrophils, EVs, NVs and HEVs. (**j**) Western blot analysis of specific markers of EVs, NVs and HEVs (n = 3). (**k**) Fluorescently labeled DFO (FAM-green) was loaded into HEVs (red). Scale bar: 1 μm. (**l**) Release curves of DFO@HEVs containing DFO. *TEM* transmission electron microscopy, *DFO* deferoxamine, *EC* endothelial cells, *Neu* neutrophil, *EVs* endothelial-derived extracellular vesicles, *NVs* neutrophil-derived extracellular vesicles, *HEVs* hybrid extracellular vesicles, *CXCR-4* C-X-C motif chemokine receptor 4, *CD11b* cluster of differentiation 11b, *FAM* 6-Carboxyfluorescein, *Dio* 3,3’-Dioctadecyloxacarbocyanine perchlorate, *Dil* 1,1’-Dioctadecyl-3,3,3,',3′-tetramethylindocarbocyanine perchlorate, *DM* diabetes mellitus

The HEVs were successfully obtained by the co-extrusion of EVs and NVs. DFO was subsequently loaded into HEVs through ultrasound and co-extrusion to produce the final DFO-loaded HEV formulation (DFO@HEVs). TEM revealed intact spherical morphologies for both HEVs and DFO@HEVs (Figure 1a). Nanoparticle tracking analysis (NTA) showed that the average particle sizes of the HEVs and DFO@HEVs were 142.1 ± 5.3 nm and 137.6 ± 5.1 nm, respectively (Figure 1b). Moreover, ζ-potential measurements confirmed that HEVs and DFO@HEVs exhibited negative surface charges of −61.52 ± 0.75 mV and − 82.36 ± 0.76 mV, respectively (Figure 1c).

In order to verify membrane fusion, DiI-labeled EVs and DiO-labeled NVs were used to track their co-localization; a simple physical mixture of the EVs and NVs was used as a control. CLSM images and co-localization analysis demonstrated membrane fusion in HEVs rather than in the physical mixture (Figure 1d–f). Fluorescence resonance energy transfer (FRET) analysis was performed using DiO-labeled EVs (donor) and Dil-labeled NVs (acceptor) to further confirm their successful fusion. The DiO-EVs alone group exhibited an emission peak exclusively at 505 nm, characteristic of DiO, under 430 nm excitation. In contrast, when the fusion of DiO-EVs and Dil-NVs at ratios of 1:1 and 1:2 (DiO-EVs:Dil-NVs) showed emission peaks corresponding to DiO (505 nm) and Dil (565 nm) under identical excitation conditions, indicating the occurrence of FRET from donor to acceptor. A strong FRET signal was detected in the HEV group, confirming membrane fusion. Importantly, only the fused vesicle group exhibited Dil emission peak under 430 nm excitation, while the simple physical mixture control showed emission exclusively in the 505 nm, characteristic of DiO, thereby further validating the successful vesicle fusion (Figure 1g, h). The protein composition of HEVs was analyzed using SDS-PAGE; the results showed a combination of protein content derived from both EVs and NVs (Figure 1i). The CXCR4 and ITGB2 membrane proteins, which are associated with vascular and inflammatory targeting, were detected using western blotting (Figure 1j). FTIR analysis of DFO@HEVs, EVs, NVs, HEVs, and DFO was performed to further confirm the successful loading of DFO on HEVs. A broad peak ranging from 3000 cm^−1^ to 3300 cm^−1^, associated with the stretching vibration of hydroxyl groups (O–H), was observed in the infrared spectra of EVs and NVs. The peak at 2937 cm^−1^ represented the asymmetric stretching vibration of C–H in methylene or methyl groups of alkyl chains. The peaks at 1651 cm^−1^ and 1541 cm^−1^ originated from the C=O stretching vibration (amide I band) and N–H bending vibration (amide II band) in amide bonds, respectively. The peak at 1230 cm^−1^ was attributed to the C–N stretching vibrations. The vibrational peak at 1070 cm^−1^ resulted from the C–O stretching vibration on the sugar ring in the EVs. The infrared spectrum of HEVs exhibited the characteristic peaks of both EVs and NVs due to their fusion. The intensities of these vibrational peaks in HEVs were higher than those in the EVs and NVs, indicating the successful preparation of HEVs. In the infrared spectrum of DFO, a broad peak ranging from 3000 cm^−1^ to 3300 cm^−1^ was attributed to the stretching vibration of O–H. The peaks at 1627 cm^−1^ and 1564 cm^−1^ originated from the C=O stretching vibration (amide I band) and N–H bending vibration (amide II band) in amide bonds, respectively. The peak at 1201 cm^−1^ was attributed to C–N stretching vibrations. A vibrational band observed at 1043 cm^−1^ resulted from the vibration of the sulfonate group, while the peak at 962 cm^−1^ was associated with N–O stretching vibration. The spectrum of DFO@HEVs consisted of the characteristic peaks similar to those of HEVs, indicating the presence of HEVs skeletal structure. Furthermore, as compared to HEVs, the vibrational peak at 1070 cm^−1^ was broader, which was due to the combined stretching vibrations of the sulfonate group and sugar ring structure. Altogether, these analyses demonstrated that the fused biofilm HEVs were successfully loaded with DFO (Figure S1b, see online supplementary material). In addition, FAM-labeled DFO (green) was successfully encapsulated into DiI-labeled HEVs (red), as observed by CLSM (Figure 1k).

The encapsulation efficiency of DFO@HEVs prepared through different methods was subsequently evaluated. The results showed that DFO@HEVs prepared through the combined ultrasound and extrusion method exhibited significantly greater drug loading (~49%) as compared to those prepared using ultrasound (~38%) or co-incubation/extrusion (~16%) alone (Figure S1c, d, see online supplementary material). Although extrusion has been reported to yield low vesicle production [46], the combined strategy applied in this study increased the encapsulation efficiency of hydrophilic drugs, such as DFO [47]. Based on the absorbance at 430 nm, corresponding to the DFO–Fe^3+^ complex, the drug release profiles were plotted in PBS and in a simulated diabetic environment *in vitro* (pH = 8 and glucose concentration = 20 mM) (Figure 1l). Notably, under high-glucose and mildly alkaline conditions, although the release of DFO was relatively slower and reached its peak concentration later, approximately 70% of the drug was released compared to normal conditions at 36 hours, and this proportion increased to around 90% by 48 hours. This demonstrates that HEVs can still maintain effective drug release concentrations even within the hyperglycemic environment. Finally, the excellent biocompatibility of DFO@HEVs was confirmed using CCK-8 assays, Calcein-AM/PI staining, and *in vitro* hemolysis testing. In brief, the endothelial cells were first treated with different concentrations of DFO and showed a significant promotion of cell proliferation at 50 μM; this was consistent with reports from previous studies (Figure S2a, see online supplementary material) [47]. Next, the endothelial cells were treated with different concentrations of DFO@HEVs and revealed that treatment with 20 μg/ml exhibited a promoting effect on cell proliferation. However, the cell activity did not further improve with the increase in concentration. Therefore, the subsequent experiments were performed using a 20 μg/ml concentration (Figure S2b, see online supplementary material). Then, the human umbilical vein endothelial cells (HUVECs) and iBMDMs were treated with 20 μg/ml of DFO@HEVs at various time points. The results showed that the best cell activity was observed at 24 hours, thus determining the optimal treatment time (Figure S2c, d, see online supplementary material). Subsequently, Calcein-AM staining and hemolysis assays confirmed the safety of DFO@HEVs (Figure S2e–g, see online supplementary material). These findings collectively demonstrated that HEVs could be successfully prepared with preserved membrane protein functionality and efficient drug encapsulation.

### Internalization and targeted uptake of DFO@HEVs

The dynamics of vesicle internalization were investigated by examining DiI-labeled HEVs under a CLSM. The results showed efficient uptake of EVs, NVs, and HEVs by HUVECs (Figure S3, see online supplementary material). The endocytic pathways were further analyzed using pharmacological inhibitors, revealing distinct internalization mechanisms: EVs primarily entered cells via clathrin-mediated endocytosis, NVs relied on lipid raft-mediated pathways, while HEVs and DFO@HEVs adopted a combined mechanism involving both clathrin-dependent and lipid raft-associated routes (Figure S4a, b, see online supplementary material).

**Figure 2:**
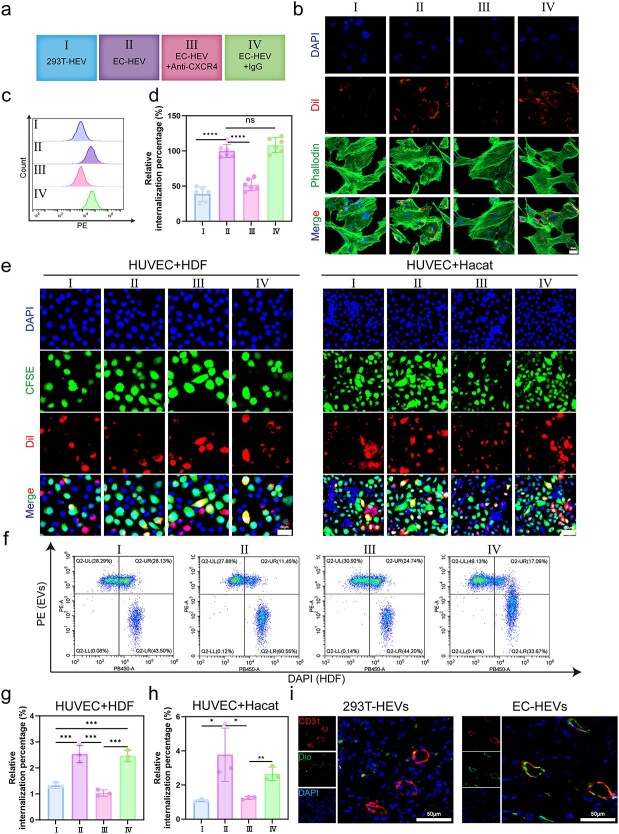
Internalization and targeted uptake of DFO@HEVs. (**a**) Grouping information. (**b**) Fluorescence images of the cellular uptake of 293T-HEVs, HEVs, HEVs pretreated with anti-CXCR4 and HEVs pretreated with IgG by HUVECs at 6 hours. Scale bar: 10 μm. (**cd**) Quantitative results of cellular uptake by HUVECs (c) and (d) FCM. Representative histograms and quantified results (n = 6). (**e**) Validation of the targeted delivery of vesicles in a cutaneous cell model *in vitro*. Scale bar: 50 μm. (**f**) Flow ctyometry and (**gh**) representative quantified results of the internalization ratio(n = 3). (**i**) Immunofluorescence image of diabetic wounds on Day 4. Dio-labeled HEVs and endothelial cells (CD31) were stained green and red, respectively (n = 3). Scale bar: 50 μm. The data are displayed as the mean ± SD. The data were assessed via one-way ANOVA and Tukey’s post hoc test; ^*^P < 0.05, ^**^P < 0.01, ^***^P < 0.001, ^****^P < 0.0001. *HEV* hybrid extracellular vesicles, *EC* endothelial cells, *Anti-CXCR4* Anti C-X-C motif chemokine receptor 4 Antibody, *IgG* immunoglobulin G, *DAPI* 4′,6-Diamidino-2-phenylindole

In order to evaluate whether CXCR4 contributed to the endothelial-targeting specificity of the EV component within HEVs, comparative uptake experiments were conducted using two types of hybrid vesicles: HEVs composed of EVs and NVs and control hybrids derived from 293T cells and NVs (293T-HEVs). HEVs were pre-incubated with either CXCR4-specific antibodies or isotype-matched IgG controls for antibody blockade experiments. Flow cytometry and fluorescence microscopy revealed significantly enhanced endothelial uptake of HEVs as compared to 293T-HEVs, while the CXCR4 blockade markedly reduced HEV internalization by HUVECs (Figure 2a–d). This confirmed the crucial role of EVs-associated CXCR4 in targeted delivery.

The endothelial targeting specificity of EVs was further validated by establishing a co-culture model. For this model, the CFSE-labeled endothelial cells were co-cultured with either DAPI-stained human dermal fibroblasts (HDFs) or keratinocytes (HaCaT cells). CLSM imaging revealed preferential accumulation of EVs in endothelial cells, with significantly less uptake by HDFs or HaCaT cells as compared to HUVECs. Notably, pretreatment with a CXCR4-neutralizing antibody significantly reduced this endothelial cell-selective uptake (Figure 2e). Flow cytometry analysis confirmed these findings: endothelial cells exhibited markedly higher EVs uptake than HDFs cells did (Figure 2f–h), reconfirming the CXCR4-dependent targeting mechanism. Collectively, these results suggested that EVs were selectively guided to endothelial cells via CXCR4, providing a strong rationale for their application in targeted vascular therapy. Finally, the vascular targeting capability of EC-HEVs was validated *in vivo* by comparing EC-HEVs and 293T-HEVs. Following wound treatment with EC-HEVs, numerous Dio-labeled HEVs colocalized with red fluorescence-labeled blood vessels, indicating that EC-HEVs exhibited superior vascular targeting ability *in vivo* (Figure 2i, Figure S5, see online supplementary material). In addition, the retention time of DiR-labeled DFO@HEVs and free DiR (Free-DiR) in mouse wounds was compared using *in vivo* imaging. The results showed that DiR-DFO@HEVs remained in the wound for a longer duration as compared to Free-DiR. DFO@HEVs were still detectable on Day 14, while only weak signals of Free-DiR were observed at the same time point, with a more rapid decline in signal intensity. This might be attributed to the superior targeting ability of DFO@HEVs toward vascular endothelium, which helped in avoiding rapid clearance. Meanwhile, it was worth noting that after subcutaneous administration of DFO@HEVs, only weak signals were detected in the liver on Days 3, 7, and 14, with no significant accumulation observed in other organs, indicating minimal impact on other tissues (Figure S6a–c, see online supplementary material).

### Inflammation-targeted delivery and modulation of neutrophil adhesion by DFO@HEVs

**Figure 3:**
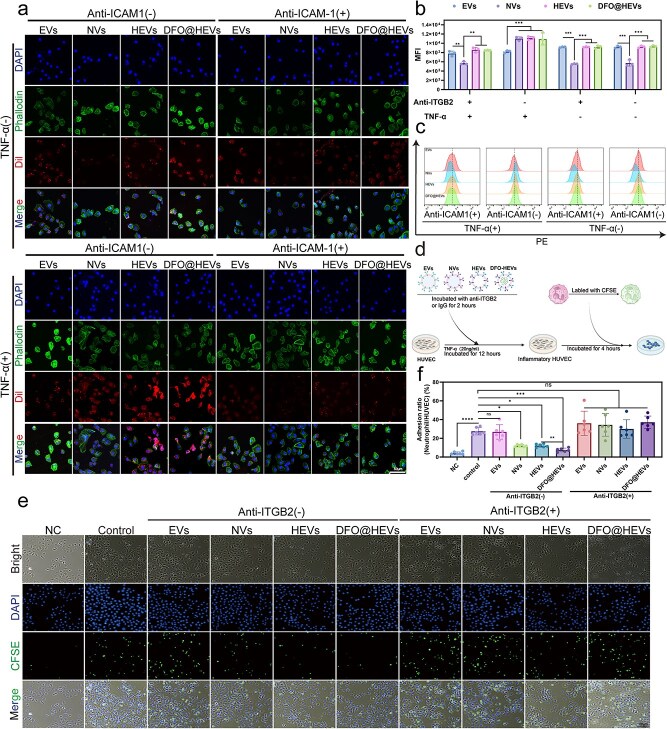
Inflammation-targeted delivery and neutrophil adhesion modulation by DFO@HEVs. (**a**) Fluorescence images of HUVECs taking up hybrid vesicles and neutrophils after hybrid vesicles were pretreated with or without anti-β2 integrin antibody. Scale bar: 50 μm. (**b**，**c**) Quantitative results of the cellular uptake of HUVECs after 6 hours, as determined by FCM (n = 3). (**d**) Schematic diagram of the experiments in (e). (**e**) Fluorescence images of neutrophil adhesion with HUVECs after hybrid vesicles were pretreated with or without an anti-β_2_integrin antibody. Scale bar: 50 μm. (**f**) Results of the quantification of neutrophil adhesion to HUVECs (n = 6). The data are displayed as the mean ± SD. The data were assessed via one-way ANOVA and Tukey’s post hoc test, ^*^P < 0.05, ^**^P < 0.01, ^***^P < 0.001, ^****^P < 0.0001. *Anti-ICAM1* anti-intercellular adhesion molecule 1 antibody, *EVs* endothelial-derived extracellular vesicles, *NVs* neutrophil-derived extracellular vesicles, *HEVs* hybrid extracellular vesicles, *DFO@HEVs* DFO-loaded hybrid extracellular vesicles, *TNF-α* tumor necrosis factor-alpha, *CFSE* Carboxyfluorescein diacetate succinimidyl ester, *Anti-ITGB2* anti β2 integrin antibody, *DAPI* 4′,6-Diamidino-2-phenylindole, *CFSE* carboxyfluorescein succinimidyl ester

The inflammation-targeting capacity of HEVs was evaluated by comparing their uptake by TNF-α-activated and non-activated control endothelial cells. Flow cytometry and confocal microscopy showed that HEVs exhibited significantly increased internalization in inflamed cells. Competitive inhibition assays using pretreatment with anti-ICAM-1 or anti-ITGB2 antibodies demonstrated abolished inflammation-specific uptake, confirming that HEV targeting relied on the ITGB2-ICAM-1 interactions. Control vesicles lacking neutrophil-derived membrane components showed no preferential targeting, highlighting the role of hybrid membrane engineering for targeted delivery to the inflammation site. Notably, HEVs maintained superior cellular uptake compared to NVs even under antibody interference, suggesting a synergistic role of the CXCR4 and ICAM-1 pathways (Figure 3a–c, Figure S7, see online supplementary material).

In order to assess the role of HEVs in modulating pathological neutrophil-endothelial interactions, an *in vitro* inflammation model was established using TNF-α-stimulated endothelial cells. The regulatory effects of four vesicle formulations, including EVs, NVs, HEVs, and DFO@HEVs, on neutrophil adhesion were systematically evaluated (Figure 3d). The results revealed that HEVs and NVs markedly reduced neutrophil adhesion to the inflamed endothelium; this effect was abolished by pre-treatment with anti-ICAM1 or anti-ITGB2 antibody. These findings demonstrated that vesicular ITGB2 components mediated competitive inhibition of neutrophil-endothelial binding. Importantly, DFO@HEVs retained full anti-adhesive efficacy, confirming that DFO encapsulation did not compromise functional integrity (Figure 3e, f; Figure S8, see online supplementary material). In order to evaluate the anti-inflammatory potential of extracellular vesicles, different vesicle formulations, including NVs, HEVs derived from the fusion of 293T-EVs and NVs (293T-HEVs), and HEVs derived from the fusion of EVs and NVs (NV-HEVs), were incubated with solutions containing either TNF-α or IL-6 at a concentration of 600 pg/ml for 24 hours. Following ultracentrifugation to remove vesicles, the inflammatory cytokine concentrations were measured. The results demonstrated that both NVs and NV-HEVs could effectively reduce TNF-α and IL-6 levels in the solution, while 293T-HEVs exhibited no substantial adsorption capacity. This highlighted the critical role of neutrophil-derived vesicles in neutralizing pro-inflammatory mediators, further supporting the therapeutic effects of NV-HEVs in modulating inflammatory microenvironments (Figure S9, see online supplementary material).

### Efficacy of DFO@HEVs in promoting cell proliferation, migration, and viability in the diabetic microenvironment *in vitro*

**Figure 4:**
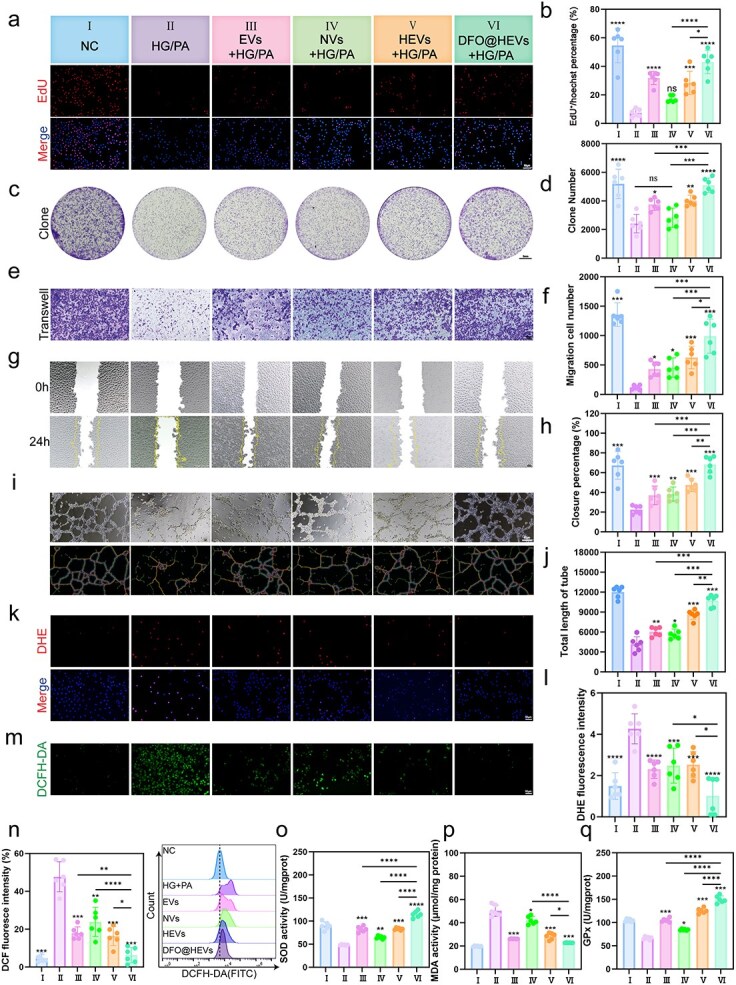
Efficacy of DFO@HEVs on cell proliferation, migration, and viability in the diabetic microenvironment *in vitro*. (**a**) Grouping information and representative images of proliferative HUVECs via EdU^+^ staining and (**b**) corresponding statistical analysis of EdU^+^ fluorescence-positive cells (n = 6). Scale bar: 100 μm. (**c**) Representative images of the HUVEC colony formation assay in a 6-well culture plate and (**d**) corresponding statistical analysis of the number of clones (n = 6). Scale bar: 5 mm. (**e**) Representative images of proliferative HUVECs in transwells and (**f**) corresponding statistical analysis (n = 6). (**g**) Scratch test results of HUVECs treated with different vesicles at 0 hour and 24 hours, and (**h**) corresponding statistical analysis of the wound healing rate (n = 6). Scale bar: 100 μm. (**i**) Representative images of the HUVEC tube formation test and (**j**) corresponding statistical analysis (n = 6). Scale bar: 100 μm. (**k**) DHE staining analysis of HUVECs and (**l**) corresponding statistical analysis of the relative fluorescence intensity of DHE (n = 6). Scale bar: 100 μm. (**m**) DCFH-DA staining analysis of HUVECs and (**n**) corresponding statistical analysis and FCM of the relative fluorescence intensity of DCF (n = 6). Scale bar: 100 μm. (**oq**) SOD activities, MDA levels and GPx activities in endothelial cells after different treatments (n = 6). The data are displayed as the mean ± SD. The data were assessed via one-way ANOVA and Tukey’s post hoc test, and the significance markers above the bars (^*, **, ***^) indicate comparisons with group II data (asterisks: ^*^P < 0.05, ^**^P < 0.01, ^***^P < 0.001, ^****^P < 0.0001). Additional horizontal brackets with corresponding symbols denote significant differences between other groups as indicated. *EVs* endothelial-derived extracellular vesicles, *NVs* neutrophil-derived extracellular vesicles, *HEVs* hybrid extracellular vesicles, *DFO@HEVs* DFO-loaded hybrid extracellular vesicles, *HG/PA* high glucose and high palmitic acid, *EdU* 5-ethynyl-2′-deoxyuridine *DHE* dihydroethidium, *DCFH-DA* 2,',7′-dichlorodihydrofluorescein diacetate, *SOD* superoxide dismutase, *MDA* malondialdehyde, *GPx* glutathione peroxidase

In order to evaluate vesicle efficacy under these conditions, HUVECs were exposed to high glucose/palmitic acid (HG/PA) and treated with various vesicles. Calcein-AM/PI staining demonstrated that vesicle treatment significantly increased the viability of HUVECs and mitigated HG + PA-induced cytotoxicity, highlighting their protective role in the diabetic microenvironment (Figure S10, see online supplementary material). EdU and clonogenic assays revealed enhanced proliferation across the vesicle-treated groups, with DFO@HEVs exhibiting the most pronounced restorative effects (Figure 4a–d). Transwell and scratch assays further demonstrated that, as compared to HG/PA control treatment, vesicle treatment significantly improved HUVEC migration, thereby accelerating wound closure (Figure 4e–h). Tube formation assays were performed to confirm functional recovery; the results showed that DFO@HEVs could restore the angiogenic capacity of HUVECs, suggesting their potential for vascular regeneration (Figure 4i, j).

Oxidative stress mitigation was evaluated using DCFH-DA and dihydroethidium (DHE) fluorescent probes, which revealed reduced ROS levels in all the vesicle-treated groups. DFO@HEVs exhibited the strongest antioxidant effect, which might be due to the iron-chelating activity of DFO (Figure 4k–n). SOD/GPx enzyme activity assays and MDA quantification revealed consistent findings, where DFO@HEVs demonstrated superior oxidative damage suppression (Figure 4o–q).

### Single-cell RNA-seq revealed antioxidant and immunoregulatory mechanisms of endothelial and macrophages in diabetic foot ulcers

**Figure 5:**
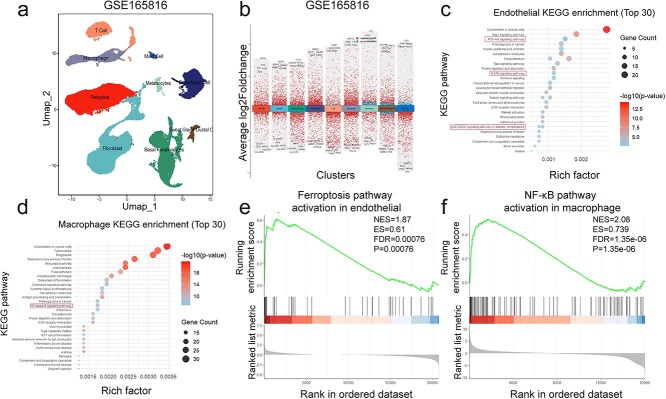
Sc-RNA-seq reveals antioxidant and immunoregulatory mechanisms of endothelial and macrophages in diabetic foot ulcers. (**a**) UMAP plot showing major cell clusters. (**b**) Cluster-specific DEGs across cell types. (**c**) The top 30 KEGG pathways enriched in endothelial cells. (**d**) The top 30 KEGG pathways enriched in macrophages. (**e**) GSEA of the ferroptosis pathway in endothelial cells (NES = 1.87, ES = 0.61, FDR = 0.00076, *P* = 0.00076). (**f**) GSEA of the NF-κB pathway in macrophages (NES = 2.08, ES = 0.739, FDR = 1.35e−06, *P* = 1.35e−06). *KEGG* Kyoto encyclopedia of genes and genomes, *NF-κB* nuclear factor-kappa B, *GSEA* gene set enrichment analysis

To delineate cell type-specific transcriptional alterations in DFUs, single-cell RNA-seq (scRNA-seq) analysis was performed on dataset GSE165816. UMAP visualization displayed distinct clustering of major cutaneous cell types, including endothelial cells, macrophages, fibroblasts, pericytes, and T cells (Figure 5a). Differential expression analysis identified cluster-specific marker genes (Figure 5b). KEGG pathway enrichment analysis uncovered significant differences among cell types. Specifically, the genes involved in the PI3K-AKT, AGE-RAGE, and MAPK signaling pathways were significantly enriched in endothelial cells, implicating oxidative stress, aberrant cell proliferation, and ferroptosis in DFU pathogenesis. GSEA further validated a significant activation of the ferroptosis pathway (NES = 1.87, ES = 0.61, FDR = 0.00076), underscoring a pivotal role of ferroptosis in endothelial dysfunction. Conversely, in macrophages, KEGG pathway enrichment analysis highlighted prominent activation of inflammation- and immunity-related pathways, especially the NF-κB signaling pathway; this was robustly confirmed by GSEA (NES = 2.08, ES = 0.739, FDR = 1.35e−06) (Figure 5c–f), indicating a pronounced pro-inflammatory phenotype. Together, these results demonstrated the distinct pathogenic mechanisms contributing to vascular and immune dysregulation in the diabetic wound microenvironment.

### DFO@HEVs suppress ferroptosis and activate PI3K-AKT signaling in the diabetic microenvironment

**Figure 6:**
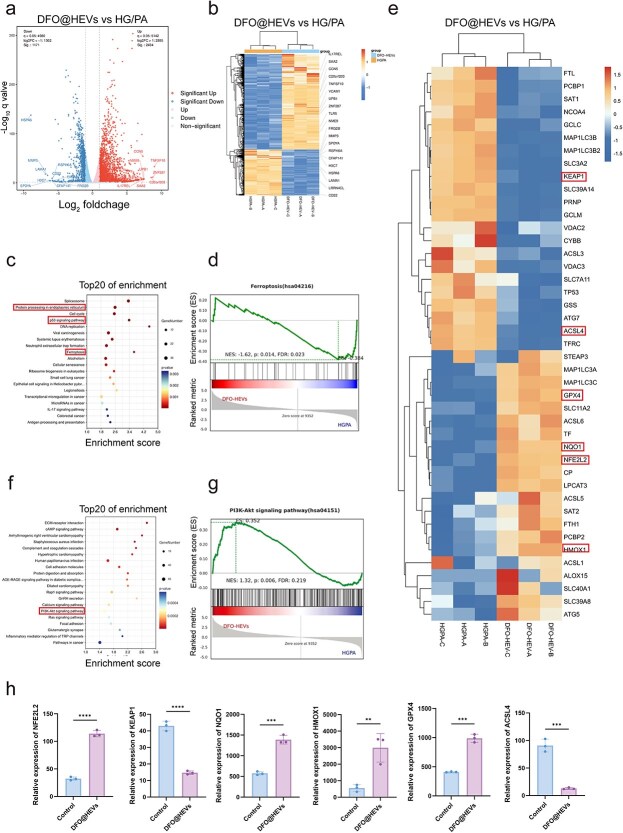
Transcriptomic analysis of DFO@HEVs-treated HUVECs under diabetic conditions. (**a**) Volcano plot of DEGs (HG/PA *vs* HG/PA + DFO@HEVs; |log2FC| > 1, FDR < 0.05). Red/blue dots denote upregulated/downregulated genes. (**b**) Heatmap of DEGs (rows: genes; columns: samples). (**c**) Top downregulated KEGG pathways in DFO@HEVs group, highlighting ferroptosis-related pathways. (**d**) GSEA showing suppressed ferroptosis in DFO@HEVs-treated cells. (**e**) Heatmap of ferroptosis-related gene expression (normalized counts). (**f**) Top upregulated KEGG pathways, including PI3K-AKT signaling. (**g**) GSEA confirming PI3K-AKT pathway activation. Data are representative of three biological replicates. *KEGG* kyoto encyclopedia of genes and genomes, *HG/PA* high glucose and high palmitic acid, *DFO-HEVs* DFO-loaded hybrid extracellular vesicles, *PI3K-AKT* phosphatidylinositol 3-kinase-protein kinase B, *NFE2L2* nuclear factor erythroid 2-related factor 2, Keap1 kelch-like ech-associated protein 1, *NQO1* nadph quinone oxidoreductase, *HMOX1* heme oxygenase 1, *GPX4* glutathione peroxidase 4, *ACSL4* acyl-coa synthetase long chain family member 4

Based on the clinical observation of ferroptosis activation in DFU endothelial cells in Figure 5, this pathway was experimentally validated, and a potential therapeutic intervention strategy was explored. Transcriptomic sequencing was performed on HUVECs exposed to a diabetic microenvironment (HG/PA) with or without DFO@HEVs treatment. Volcano plot analysis identified 3605 differentially expressed genes (DEGs) (|log2FC| >1, FDR <0.05) (Figure 6a). Hierarchical clustering confirmed distinct transcriptional reprogramming induced by DFO@HEVs (Figure 6b). KEGG pathway enrichment analysis revealed a significant downregulation of ferroptosis-associated pathways, including ferroptosis per se, p53, MAPK, and endoplasmic reticulum stress (Figure 6c). GSEA further validated this suppression, showing negative enrichment for ferroptosis in DFO@HEVs-treated cells (NES = −1.62, FDR = 0.023) (Figure 6d). Consistently, heatmap analysis demonstrated upregulation of key antioxidant genes (*GPX4, FTH1, HMOX1, NQO1*, and *NFE2L2*) and downregulation of pro-ferroptotic markers (*ACSL4* and *NCOA4*) (Figure 6e). In parallel, PI3K-AKT signaling was markedly activated (Figure 6f), as supported by GSEA (NES = 1.32, FDR = 0.219) (Figure 6g). Quantitatively analyzing the relative expression levels of key ferroptosis-related genes confirmed that DFO@HEVs significantly upregulated antioxidant genes while suppressing pro-ferroptotic genes (Figure 6h). These findings demonstrated that DFO@HEVs alleviated diabetic endothelial injury by inhibiting ferroptosis and activating the PI3K-AKT/Nrf2 axis.

### DFO@HEVs promote antioxidant responses by activating PI3K/AKT/Nrf2 pathway

The transcriptomic findings (Figures 5, 6) revealed the activation of ferroptosis and apoptosis in endothelial cells from DFU tissue. Next, the functional relevance of these transcriptional signatures was experimentally validated. Emerging evidence suggests that the diabetic microenvironment can trigger endothelial ferroptosis via iron overload and lipid peroxidation [48–50]. Thus, ferroptosis suppression might be a promising therapeutic strategy for chronic wounds.

**Figure 7:**
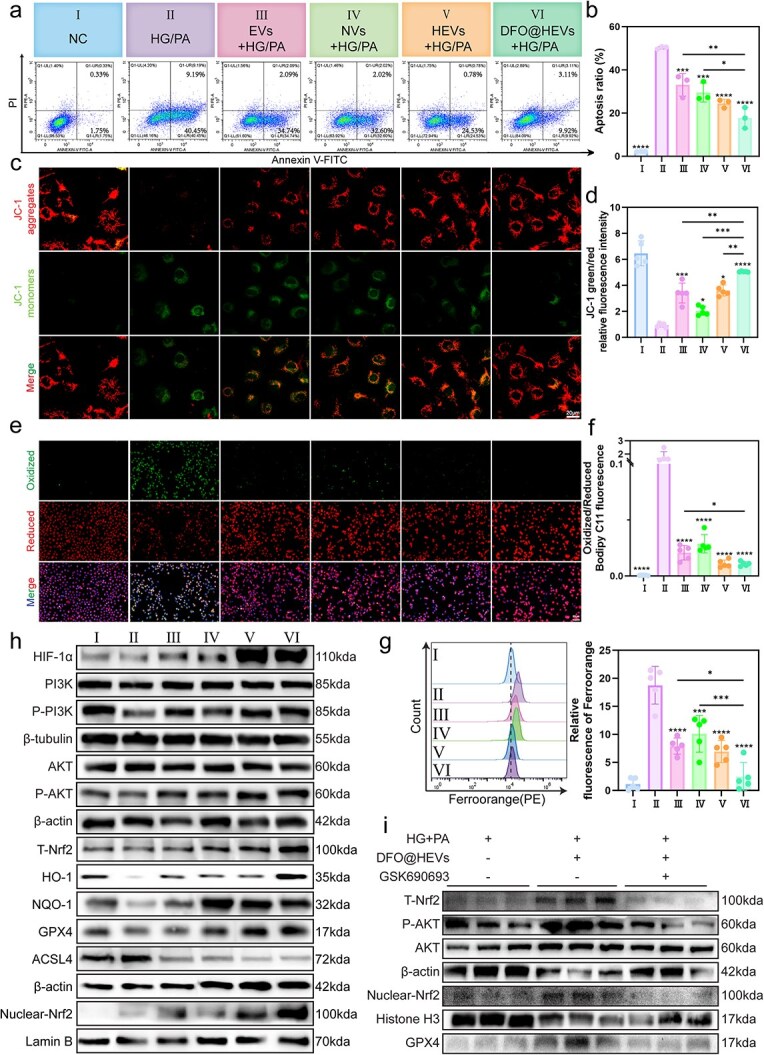
DFO@HEVs reprogram diabetic wound healing via a ferroptosis-inhibiting PI3K/AKT/Nrf2 circuit. (**a**) Grouping information and flow cytometry analysis of cell apoptosis in each group and (**b**) quantitative analysis of the apoptosis rate (n = 3). (**c**) JC-1 staining analysis of HUVECs and (**d**) corresponding statistical analysis of the relative fluorescence intensity of JC-1 (n = 5). Scale bar: 20 μm. (**e**) Representative fluorescence images and quantitative analysis (**f**) of lipid peroxidation levels in HUVECs treated with different vesicles (n = 5). Scale bar:50 μm. (**g**) Quantitative analysis and flow cytometry of ferroptosis levels in HUVECs treated with different vesicles (n = 5). (**h**) Western blot of HIF-1α, PI3K, P-PI3K, AKT, P-AKT, nuclear NRF2, total NRF2, NQO1, HO-1, GPX4W and ACSL4 in HUVECs after treatment (n = 3). (**i**) Western blot analysis of endothelial cells after treatment with GSK690693 (n = 3). The data are displayed as the mean ± SD. The data were assessed via one-way ANOVA and Tukey’s post hoc test, and the significance markers above the bars (^*, **, ***^) indicate comparisons with group II data (asterisks: ^*^P < 0.05, ^**^P < 0.01, ^***^P < 0.001, ^****^P < 0.0001). Additional horizontal brackets with corresponding symbols denote significant differences between other groups as indicated. *PI* propyl iodide, *HIF-1α* hypoxia inducible factor 1 subunit alpha, *PI3K* phosphatidylinositol 3-kinase, *P-PI3K* phosphorylated phosphatidylinositol 3-kinase, *AKT* protein kinase b, *P-AKT* phosphorylated protein kinase b, *Nrf2* nuclear factor erythroid 2-related factor 2, *NQO1* nadph quinone oxidoreductase, *HO-1* heme oxygenase 1, *GPX4* glutathione peroxidase 4, *ACSL4* acyl-coa synthetase long chain family member 4

In order to assess this, mitochondrial function and apoptosis were evaluated in HUVECs exposed to HG/PA. JC-1 staining, TMRE flow cytometry, and Annexin V/PI assays demonstrated that DFO@HEVs could significantly restore the mitochondrial membrane potential and reduce apoptosis under diabetic stress (Figure 7a–d, Figure S11, see online supplementary material). C11-BODIPY and FerroOrange probes were used to measure lipid ROS and labile iron levels, respectively. The results showed marked reductions in their levels following DFO@HEV treatment, confirming the effective inhibition of ferroptosis (Figure 7e–g, Figure S12, see online supplementary material).

The transcriptomic data showed enrichment of PI3K/AKT and Nrf2-related pathways in endothelial cells in Figure 6. Therefore, these signaling networks were further explored. Western blot analysis showed increased phosphorylation of PI3K and AKT, along with HIF-1α stabilization, confirming the activation of the PI3K/AKT pathway by DFO@HEVs. Simultaneously, consistent with the scRNA-seq analysis, Nrf2 was translocated into the nucleus, and the antioxidant protein levels, including HO-1, NQO1, and GPX4, were upregulated. In addition, DFO@HEVs significantly suppressed ACSL4, a key ferroptosis-related protein (Figure 7h), indicating their dual role in alleviating oxidative stress and inhibiting ferroptosis.

In order to assess the functional importance of the PI3K/AKT pathway, the AKT inhibitor GSK690693 was used. As shown in Figure 7i, the AKT inhibition reversed the protective effects of DFO@HEVs. It blocked Nrf2 activation and GPX4 upregulation, and diminished the suppression of ferroptosis. Moreover, GSK690693 impaired the ability of DFO@HEVs to promote the migration, proliferation, and tube formation of endothelial cells (Figures S13–S18, see online supplementary material). These results collectively highlighted the PI3K/AKT pathway as central to DFO@HEV-induced angiogenesis and ferroptosis protection.

### Immunomodulatory effects of DFO@HEVs on macrophage polarization and inflammation

Next, their immunomodulatory potential was further explored. The scRNA-seq analysis of diabetic wound tissues (Figure 5) revealed that inflammation-related pathways were significantly enriched in macrophage subsets, with the NF-κB signaling pathway emerging as a dominant node. NF-κB activation is a central mediator of M1 polarization and inflammatory cytokine production, and its aberrant activation contributes to sustained macrophage dysfunction and chronic tissue damage. Based on these scRNA-seq analysis results, the NF-κB axis was selected for the downstream validation of DFO@HEVs-mediated immune reprogramming.

**Figure 8:**
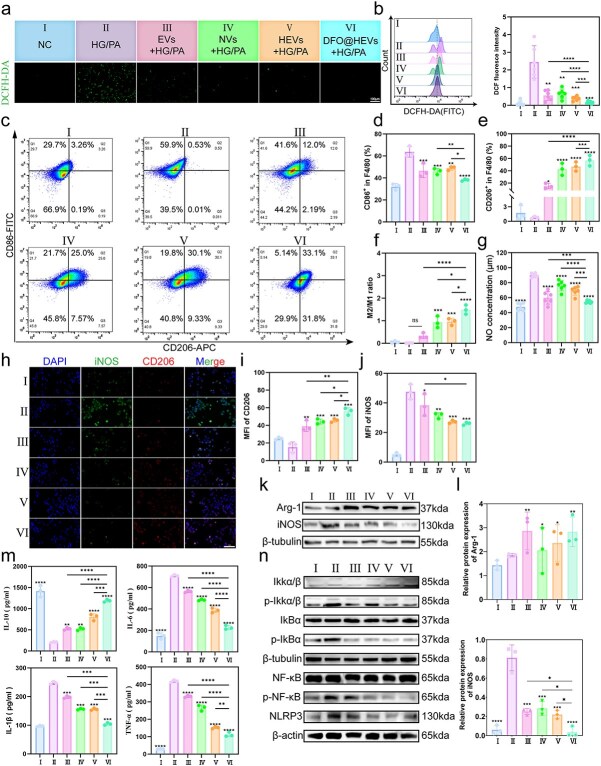
Immunomodulatory effects of DFO@HEVs on macrophage polarization and inflammation. (**a**) Fluorescence images and (**b**) quantitative expression of ROS after treatment in inflamed iBMDMs (n = 6).Scale bar: 50 μm. (**c**) Investigation of the phenotypic polarization of macrophages. FCM was used to detect the polarization of inflamed iBMDMs after treatment with different vesicles, and iBMDMs stimulated without LPS served as the normal control group (n = 3). (**d**) CD86- and (**e**) CD206-positive cells indicate the M1 and M2 subtypes, respectively, after treatment. (**f**) Quantitative representation of the M2/M1 ratio after treatment. (**g**) Production of NO after treatment of inflamed macrophages (n = 6). (**h**) Representative immunofluorescence images and (**ij**) quantitative expression of iNOS and CD206 after treatment of inflamed iBMDMs (n = 3). Scale bar: 100 μm. (**k**) Western blot analysis of Arg-1 and iNOS in iBMDMs after different treatments and (**l**) corresponding statistical analysis (n = 3). (**m**) TNF-α, IL-10 and IL-6 levels in the supernatant of inflamed macrophages after treatment (n = 3). (**n**) Western blot analysis of Iκkα/β, P-Iκkα/β, IκBα, P-IκBα, NF-κB, P-NF-κB and NLRP3 in iBMDMs after different treatments (n = 3). The data are displayed as the mean ± SD. The data were assessed via one-way ANOVA and Tukey’s post hoc test, and the significance markers above the bars (^*, **, ***^) indicate comparisons with group II data (asterisks: ^*^P < 0.05, ^**^P < 0.01, ^***^P < 0.001, ^****^P < 0.0001). Additional horizontal brackets with corresponding symbols denote significant differences between other groups as indicated. *DCFH-DA* 2′,7′-dichlorodihydrofluorescein diacetate, *CD86* cluster of differentiation 86, *CD206* cluster of differentiation 206, *iNOS* inducible nitric oxide synthase, *Arg-1* arginase-1, *IL* interleukin, *TNF-α* tumor necrosis factor-alpha, *Iκk* inhibitor of nuclear factor kappa-b kinase, *IκB* inhibitor of nuclear factor kappa-b, *NF-κB* nuclear factor kappa-b, *NLRP3* nod-like receptor family pyrin domain containing 3

The macrophages exposed to HG/PA conditions exhibited DFO@HEVs marked reductions in intracellular ROS levels (Figure 8a, b), indicating effective suppression of upstream redox-driven inflammatory signaling. Flow cytometric analysis of canonical surface markers (CD86 and CD206) revealed a phenotypic shift toward an anti-inflammatory M2 profile, accompanied by reduced M1 populations (Figure 8c–f). This polarization transition was further substantiated by a decrease in NO secretion (Figure 8g). Moreover, immunofluorescence staining further revealed decreased iNOS and increased CD206 expression (Figure 8h–j). Western blot analysis confirmed the downregulation of iNOS and upregulation of arginase-1 (Figure 8k, l). This was consistent with functional reprogramming toward an anti-inflammatory macrophage phenotype.

At the molecular level, DFO@HEVs reduced the phosphorylation of IKKα/β, preserved IκBα levels, and decreased the nuclear translocation of NF-κB p65, thereby suppressing the key inflammatory NF-κB pathway (Figure 8m). The NLRP3 inflammasome expression was also notably downregulated (Figure 8n), indicating that DFO@HEVs could alleviate both inflammatory initiation and effector responses.

Together, these results demonstrated that DFO@HEVs could modulate macrophage function by restoring redox balance, inhibiting NF-κB signaling, and promoting M2 polarization. In combination with their previously observed protective effects on endothelial cells, these findings highlighted the ability of DFO@HEVs to reshape the vascular-immune microenvironment in diabetic wounds.

In order to further examine whether macrophage reprogramming by DFO@HEVs functionally supported blood vessel repair, endothelial cells were cultured with conditioned media from treated macrophages. The conditioned media from DFO@HEV-treated macrophages significantly improved migration and tube formation of endothelial cells (Figures S19, S20, see online supplementary material). This indicated that the immunomodulatory effects of DFO@HEVs could enhance angiogenesis through paracrine signaling.

### DFO@HEVs promote efferocytosis in macrophages

**Figure 9:**
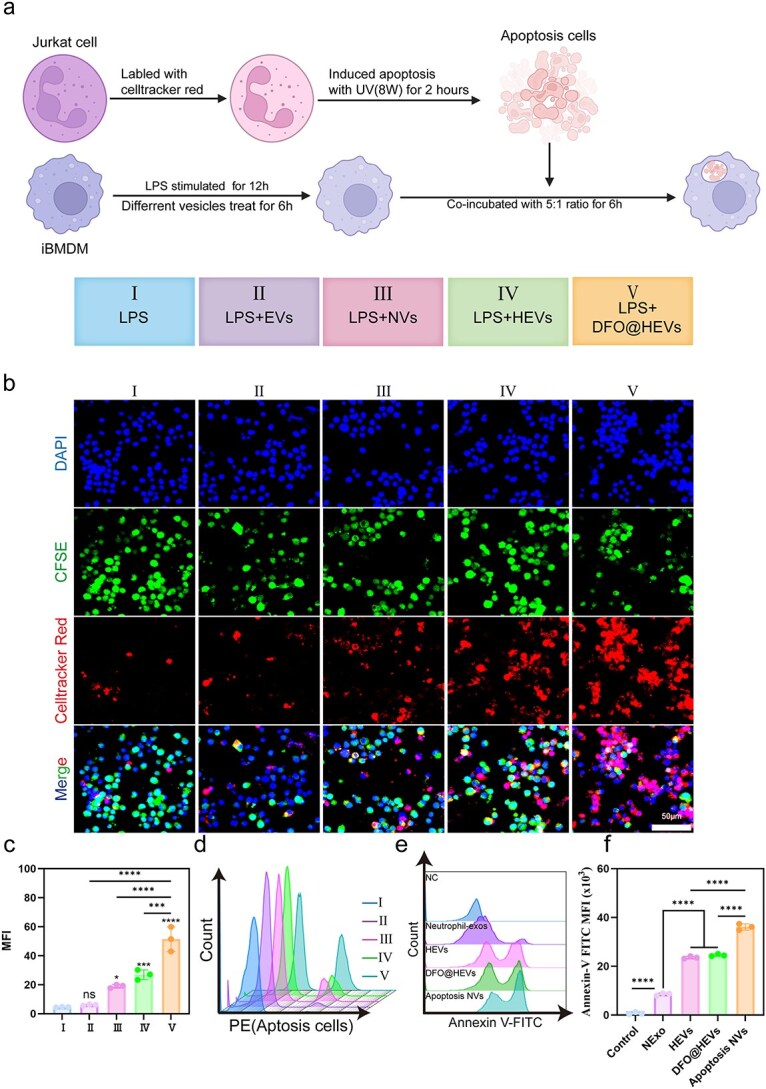
Promotion of efferocytosis by DFO@HEVs. (**a**) Schematic illustration of the experiments in (**b**), and the grouping information. (b) CLSM image analysis to evaluate efferocytosis *in vitro*. iBMDMs were labeled with CFSE in green, apoptotic Jurkat cells in red, and the nuclei of iBMDMs in blue. Scale bar: 50 μm. (**c**) Corresponding quantified results and (**d**) FCM (n = 3). (**ef**) Flow cytometric analysis of Annexin-V positivity in NC HUVECs, neutrophil-exos, HEVs, DFO-HEVs and apoptotic NVs internalized by HUVECs (n = 3). The data are displayed as the mean ± SD. The data were assessed via one-way ANOVA and Tukey’s post hoc test, ^*^P < 0.05, ^**^P < 0.01, ^***^P < 0.001, ^****^P < 0.0001. *iBMDM* immortalized bone marrow-derived macrophages, *LPS* lipopolysaccharide, *EVs* endothelial-derived extracellular vesicles, *NVs* neutrophil-derived extracellular vesicles, *HEVs* hybrid extracellular vesicles, *DFO@HEVs* DFO-loaded hybrid extracellular vesicles, *NExo* neutrophill-derived exosomes, *DAPI* 4′,6-Diamidino-2-phenylindole, *CFSE* carboxyfluorescein succinimidyl ester

In order to evaluate the therapeutic effects of efferocytosis, a co-culture model of CFSE-labeled apoptotic Jurkat cells and conditioned macrophages was established (Figure 9a). Confocal imaging demonstrated that DFO@HEV treatment could significantly augment phagocytic activity, as evidenced by the robust uptake of fluorescent apoptotic cells compared to that in the untreated or injury model groups. While EVs, NVs, and HEVs showed moderate improvement, flow cytometry confirmed the superior efficacy of DFO@HEVs in promoting apoptotic cell clearance (Figure 9b–d).

Further mechanistic investigations focused on phosphatidylserine (PS) surface expression, a key ‘eat-me’ signal for efferocytosis [51]. Annexin V-FITC labeling revealed minimal PS expression in HUVECs without vesicles, while HEVs and DFO@HEVs presented PS levels comparable to those of apoptotic NVs (positive controls) (Figure 9e, f). The observed increase in PS exposure on extracellular vesicles might result from the disruption of membrane integrity during extrusion, a process that induces stochastic redistribution of inner and outer membrane components. This phenomenon aligned with prior studies demonstrating that extruded extracellular vesicles exhibited randomized membrane orientation, where inner and outer leaflets were non-specifically reconstituted [43, 52]. This PS enrichment was directly correlated with enhanced efferocytosis capacity, suggesting that vesicle-associated PS could facilitate macrophage recognition and engagement of the apoptotic cargo.

### DFO@HEVs accelerate diabetic wound healing *in vivo*

**Figure 10:**
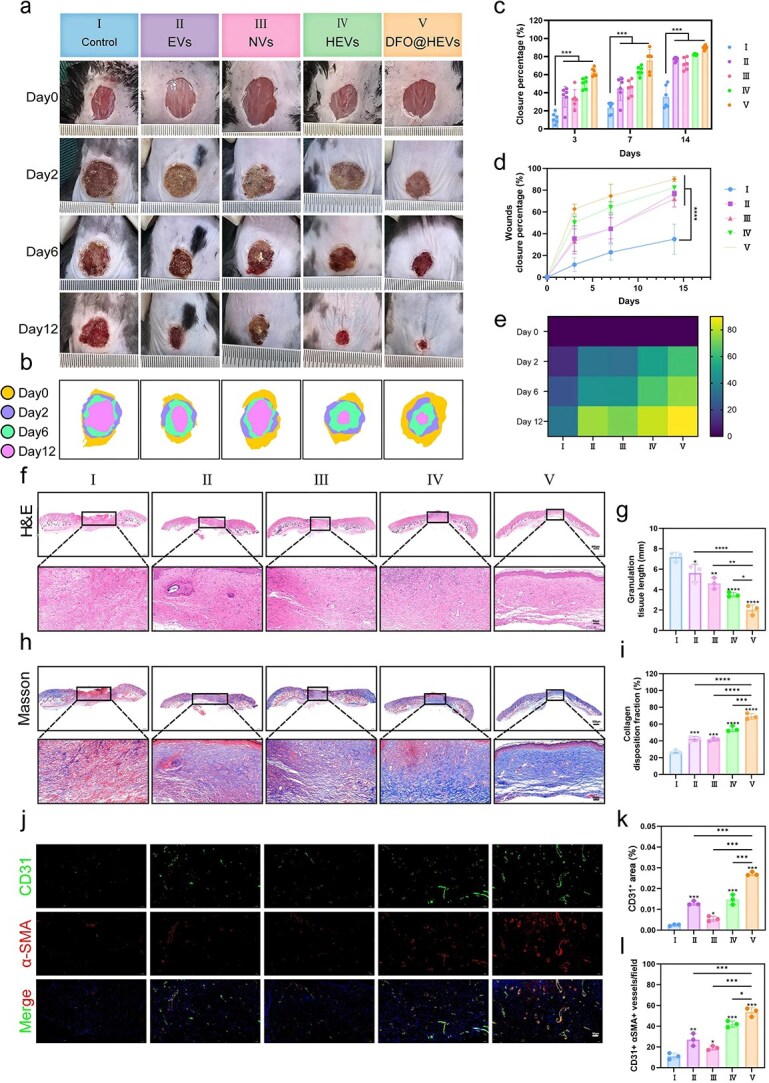
DFO@HEVs accelerate diabetic wound healing *in vivo*. (**a**) Grouping information and representative images, (**b**) monitoring and (**c**–**e**) statistics of wound closure at Days 0, 2, 6, and 12 (n = 6). (**f**–**i**) Representative images of H&E and Masson staining results on Day 12 and corresponding statistical analysis of granulation tissue length (n = 3) and collagen deposition (n = 3) in the wound tissue. Scale bar: 50 μm. (**j**–**l**) Representative images of immunofluorescence staining of CD31 and α-SMA in wound tissue and corresponding statistical analysis of the relative fluorescence intensity (n = 3). Scale bar: 50 μm. The data are displayed as the mean ± SD. The data were assessed via one-way ANOVA and Tukey’s post hoc test, and the significance markers above the bars (^*, **, ***^) indicate comparisons with group II data (asterisks: ^*^P < 0.05, ^**^P < 0.01, ^***^P < 0.001, ^****^P < 0.0001). Additional horizontal brackets with corresponding symbols denote significant differences between other groups as indicated. *EVs* endothelial-derived extracellular vesicles, *NVs* neutrophil-derived extracellular vesicles, *HEVs* hybrid extracellular vesicles, *DFO@HEVs* DFO-loaded hybrid extracellular vesicles, *H&E* hematoxylin and eosin, *CD31* cluster of differentiation 31, *α-SMA* alpha smooth muscle actin

Using comprehensive histopathological and molecular analyses, the therapeutic efficacy of DFO@HEVs in diabetic wound repair was systematically validated. A series of wound imaging revealed that DFO@HEVs achieved the most pronounced acceleration of wound closure among all the experimental groups, with nearly complete re-epithelialization observed in diabetic mice by Day 12 post-injury (Figure 10a). The quantitative analysis of the wound margins (Figure 10b) and statistical comparisons (Figure 10c–e) showed significantly faster contraction rates of DFO@HEVs as compared to the other vesicle formulations, further confirming their exceptional healing kinetics. Additional comparative studies evaluating the treatment efficacy of free DFO and DFO@HEVs in diabetic wound healing were conducted to further validate the therapeutic potential of DFO@HEVs for drug delivery. The results (Figure S21, see online supplementary material) demonstrated that DFO@HEVs significantly outperformed free DFO, which might be due to the prolonged retention time of DFO@HEVs within the wound area.

Histopathological evaluation highlighted the excellent regenerative capacity of DFO@HEVs. Hematoxylin and eosin (H&E)-stained sections (Figure 10f, g) revealed that DFO@HEVs could completely restore epithelium with minimal inflammatory infiltration. Moreover, Masson’s trichrome staining (Figure 10h, i) also revealed more organized collagen deposition and mature connective tissue structure in DFO@HEVs-treated wounds than in the other groups, reflecting advanced ECM remodeling. The pro-angiogenic effects of DFO@HEVs in diabetic wounds were evaluated by immunofluorescence staining for CD31 and α-SMA. The results revealed increased neovascularization and mature vessel density across all the EV-treated groups, with DFO@HEVs showing the most pronounced vascular regenerative capacity. Notably, NVs exhibited limited efficacy due to a lack of direct angiogenic activity (Figure 10j–l). Given the crucial role of fibroblasts in wound healing, Vimentin expression levels were further assessed to evaluate fibroblast activity in the wound area. The results demonstrated that although DFO@HEVs could primarily target vascular endothelial cells, they effectively restored fibroblast function in the wound region. (Figure S22, see online supplementary material). This effect might be attributed to their overall improvement effects on the wound inflammatory microenvironment.

**Figure 11:**
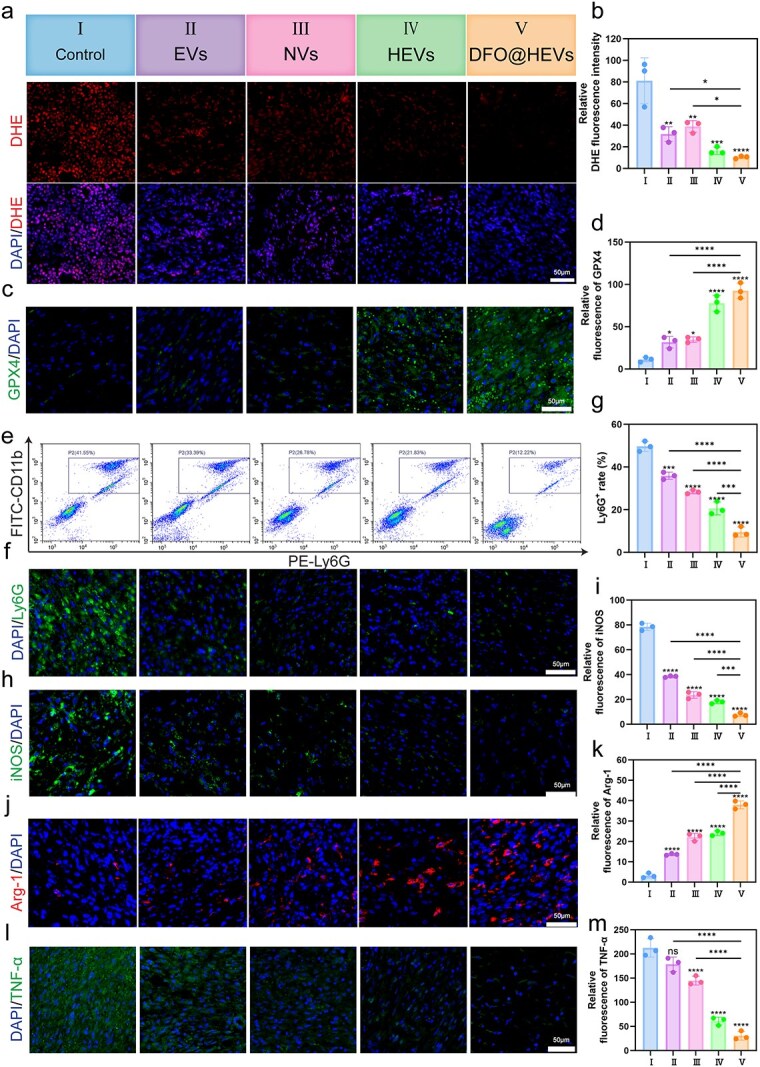
Histological and immunofluorescence analysis of diabetic wound healing. (**ab**) Grouping information and DHE staining of wound sections from each group for evaluating the ROS levels in the wound tissue on Day 12 post-treatment (n = 3). Scale bar: 50 μm. (**c**–**e**) Representative FCM plots and immunofluorescence for quantitative analysis of the neutrophil proportion in wound tissue (n = 3). Scale bar: 50 μm. (**fg**) Representative images of immunofluorescence staining of GPX4 (n = 3). Scale bar: 50 μm. (**h**–**k**) Representative images of immunofluorescence staining of iNOS and Arg-1 in wound tissue (n = 3). Scale bar: 50 μm. (**lm**) representative images of immunofluorescence staining of TNF-α (n = 3). Scale bar: 50 μm. The data are displayed as the mean ± SD. The data were assessed via one-way ANOVA and Tukey’s post hoc test, and the significance markers above the bars (^*, **, ***^) indicate comparisons with group II data (asterisks: ^*^P < 0.05, ^**^P < 0.01, ^***^P < 0.001, ^****^P < 0.0001). Additional horizontal brackets with corresponding symbols denote significant differences between other groups as indicated. *EVs* endothelial-derived extracellular vesicles, *NVs* neutrophil-derived extracellular vesicles, *HEVs* hybrid extracellular vesicles, *DFO@HEVs* DFO-loaded hybrid extracellular vesicles, *DHE* dihydroethidium, *CD11b* cluster of differentiation 11b, *Ly6G* lymphocyte antigen 6 complex locus g6d, *GPX4* glutathione peroxidase 4, *iNOS* inducible nitric oxide synthase, *Arg-1* arginase-1, *TNF-α* tumor necrosis factor-alpha, *DAPI* 4′,6-Diamidino-2-phenylindole

Based on these findings, the antioxidant, anti-ferroptosis, and anti-inflammatory effects of DFO@HEVs were further validated using *in vivo* experiments. DHE staining on Day 12 demonstrated that DFO@HEVs-treated wounds exhibited significantly reduced ROS levels; this was consistent with the *in vitro* data (Figure 11a, b). Immunofluorescence analysis of GPX4 confirmed enhanced ferroptosis resistance in the DFO@HEVs group (Figure 11c, d).

Flow cytometry and immunofluorescence analyses of wound tissues demonstrated that treatment with NVs, HEVs, and DFO@HEVs could significantly reduce neutrophil infiltration (CD11b^+^/Ly6G^+^ cells). EVs exhibited the weakest effects, which might be due to their localized oxidative stress mitigation. Ly6G immunofluorescence showed similar observations (Figure 11e–g).

Furthermore, to assess macrophage polarization dynamics, immunofluorescence was used. Consistent with the *in vitro* findings, the result demonstrated decreased iNOS expression (M1 marker) and increased Arg-1 levels (M2 marker) in the vesicle-treated groups, with the DFO@HEVs exhibiting superior M2 polarization efficacy (Figure 11h–k). Consistent with these findings, immunofluorescence analysis of TNF-α also confirmed that DFO@HEVs significantly reduced the level of inflammatory factors in the wound area on Day 7 (Figure 11l–m). Collectively, the *in vivo* results demonstrated that DFO@HEVs could accelerate diabetic wound healing by reducing neutrophil infiltration (CD11b^+^/Ly6G^+^ cells) and promoting macrophage polarization toward the anti-inflammatory M2 phenotype (increased Arg-1 and decreased iNOS). This was consistent with the improved vascular regeneration and inflammation resolution observed in treated wounds.

Finally, the biocompatibility of DFO@HEVs was estimated *in vivo*. H&E staining was performed on the main organ tissues (heart, liver, spleen, lungs, and kidneys) collected from diabetic mice with wounds on Day 14 after treatment. No significant tissue damage or obvious inflammatory lesions were observed in the different treatment groups (Figure S23, see online supplementary material), indicating that these extracellular vesicles exhibited good biocompatibility *in vivo*.

## Discussion

In recent years, chronic diabetic wounds have remained a major clinical challenge, largely due to persistent oxidative stress, unresolved inflammation, and impaired angiogenesis [1–3]. These pathological factors interact to form a vicious cycle that significantly delays wound repair and increases the risk of amputation. Conventional treatments, such as negative-pressure therapy, growth factors, or single-agent drugs, often target only one aspect of the wound pathology, resulting in limited efficacy [3, 4, 53]. Therefore, developing multi-target, synergistic therapeutic strategies is of great importance for improving diabetic wound healing.

With the rapid advancement of emerging biotechnologies, extracellular vesicles have attracted considerable attention owing to their low immunogenicity, high loading capacity, ease of engineering, and diverse biological activities. These properties make extracellular vesicles highly promising for biomedical applications, including early diagnosis, therapeutic intervention, and prognosis monitoring [25]. However, the limited targeting efficiency, low production yield, and poor drug-loading capacity of natural extracellular vesicles have significantly restricted their clinical translation [54]. Hybrid membrane extracellular nanovesicles are bioinspired nanocarriers that integrate the unique properties of multiple membrane sources, providing enhanced stability, targeting ability, and multifunctionality. Our previous study demonstrated that HEVs could be generated by fusing HUVEC-derived vesicles with liposomes, thereby retaining the functional advantages of both components [39]. In addition, other studies have reported the construction of HEVs by combining different cell types [55, 56]. Despite variable cellular sources, a common finding was that such HEVs could successfully integrate the functions of their distinct origins, resulting in more efficient delivery vehicles with enhanced therapeutic potential. Previous studies showed that EVs could promote angiogenesis, while NVs exhibited strong inflammation-homing capabilities [37–43]. The current study combined the advantages of both systems by engineering HEVs and loading them with the iron chelator DFO (DFO@HEVs). This design endowed the platform with dual-targeting ability and multifunctional therapeutic potential.

Previous studies have shown that EVs retain the homing ability of their parent cells and preferentially target endothelial tissues [57]. This targeting is mediated by the CXCR4 membrane protein, which remains on the vesicle surface following extrusion [37, 58]. Neutrophils can bind firmly to ICAM-1/2 (a vascular endothelial surface adhesion molecule) *via* ITGB2, a key mechanism that dominates the recruitment of inflammatory tendencies in immune cells [59, 60]. The NV components in HEVs enable dual therapeutic actions: (i) inflammatory endothelial targeting through ITGB2-ICAM-1 interactions for site-specific drug delivery and (ii) competitive inhibition of neutrophil adhesion by blocking ICAM-1 binding sites, thereby attenuating pathological neutrophil–endothelial interactions [61–63]. Importantly, using both *in vitro* and *in vivo* experiments, the current study confirmed that HEVs could successfully integrate these dual targeting functions of the two parent EV types. Furthermore, antibody-blocking assays verified the specific interaction sites between the vesicles and target cells *in vitro*, providing direct mechanistic evidence for their targeting capacity.

Enhancing the proliferation, migration, and viability of cutaneous cells in diabetic environments is critical for diabetic wound healing. The HG/PA model can recapitulate the multifactorial diabetic microenvironment, which includes oxidative stress, lipid toxicity, and metabolic dysregulation [64, 65]. In this study, DFO@HEVs could restore the growth, migration, and tube formation ability of HG/PA-damaged HUVECs. They also reduced intracellular ROS, improved mitochondrial membrane potential, and lowered lipid peroxidation. As compared to HEVs, DFO@HEVs showed stronger therapeutic effects, suggesting that HEVs could effectively deliver DFO into target cells. Some results showed no clear statistical difference between the two groups, which may be due to the small sample size. Notably, HEVs alone showed better performance than EVs or NVs, showing that the hybrid design could combine the advantages of both EV types. This combination overcame the limits of single vesicle sources. For example, Figure 4A–D shows that NVs had little effect on cell growth, while HEVs showed greater benefits. In Figure 8, EVs showed mild anti-inflammatory effects, and NVs showed stronger effects, while HEVs showed effects equal to the combination of both extracellular vesicles, highlighting the value of the hybrid approach.

At the molecular level, DFO@HEVs activated both the PI3K-AKT-HIF-1α and Nrf2 signaling pathways. The upregulation of HIF-1α might be driven by DFO, promoting endothelial proliferation, while the simultaneous induction of Nrf2 might depend on AKT signaling. Inhibition of AKT attenuated Nrf2 and its downstream antioxidant defense, suggesting that the protective effects of DFO@HEVs against ferroptosis were mediated through AKT-dependent stabilization of Nrf2. This was consistent with previous studies [66]. Transcriptomic analysis further supported this mechanism by demonstrating downregulation of ferroptosis-associated genes along with the activation of the PI3K-AKT signaling pathway.

Besides redox imbalance, persistent low-grade inflammation is a hallmark of diabetic wounds and a major barrier to healing. The impairment of macrophage plasticity, particularly the defective transition from M0 to the anti-inflammatory M2 phenotype, delays wound closure, reduces angiogenesis, and hampers matrix remodeling [67, 68]. Importantly, M2 macrophages possess strong efferocytosis capacity, enabling the efficient clearance of apoptotic cells to prevent secondary necrosis. This process promotes tissue homeostasis by releasing pro-resolving mediators, such as TGF-β and IL-10, which establish a self-reinforcing cycle that amplifies continual efferocytosis, suppresses inflammation, and accelerates repair [69, 70]. In chronic wounds, defective efferocytosis sustains inflammation and blocks healing, highlighting the therapeutic value of restoring this cycle [71]. Previous studies showed that DFO could promote M2 polarization via redox regulation, while NVs exerted inherent anti-inflammatory effects [43, 61, 63, 72–75]. Based on these findings, the current study demonstrated that DFO@HEVs could enhance macrophage M2 polarization and restore efferocytosis, thereby breaking the cycle of persistent inflammation.

Consistent with *in vitro* results, *in vivo* experiments in diabetic mice further confirmed the therapeutic efficacy of DFO@HEVs, which was evidenced by histological staining, immunofluorescence, and flow cytometry. These results highlighted the strong potential of DFO@HEVs as an effective strategy for promoting wound healing in diabetic ulcers.

As compared to previous single-modality approaches, the current study provided several distinct advantages. By integrating the dual-targeting features of natural vesicles with the therapeutic function of DFO, DFO@HEVs achieved efficient delivery and coordinated modulation of multiple wound-healing barriers. Importantly, HEVs successfully delivered DFO, showing targeting effects toward both vascular endothelium and inflamed sites. Moreover, they significantly prolonged drug retention in the wound area as compared to the direct administration of small-molecule compounds, thereby enhancing therapeutic efficacy. This optimized pharmacokinetic effect, in combination with the synergistic disruption of the pathological cycle comprising oxidative stress, ferroptosis, and inflammation, demonstrated better therapeutic effects of HEVs over conventional delivery carriers. These advantages highlighted the potential of biohybrid extracellular vesicles in managing complex chronic wounds. They also revealed the broad application prospects of HEVs as delivery vehicles for small-molecule compounds and nanomaterials in treating various vascular inflammatory diseases beyond diabetic wounds.

Despite these encouraging results, several limitations should be noted. First, the *in vivo* assessment was limited to murine diabetic wound models, and validation in large-animal models is required before clinical translation. Second, challenges remain in the quality control of HEVs, the determination of optimal therapeutic dosage, and the comparison between different EV types. Moreover, additional functionalization and surface modification are needed to enhance targeted delivery, particularly for diabetic wounds. It is also worth noting that hemostasis and antimicrobial activity play crucial roles in diabetic wound healing; [76] however, these aspects were not specifically addressed in the current therapeutic design. Future studies could explore the integration of extracellular vesicles with advanced biomaterials (such as hydrogels or antibacterial coatings) to achieve multifunctional synergy, thus further improving the therapeutic potential for clinical application.

In summary, this study demonstrated that DFO@HEVs act as multifunctional nanotherapeutics that could simultaneously promote angiogenesis, suppress ferroptosis, and resolve chronic inflammation in diabetic wounds. By successfully delivering DFO and leveraging their dual-targeting capacity, HEVs provided a promising foundation for next-generation therapies in wound management and might broaden their translational potential to treat other vascular inflammatory disorders.

## Conclusions

This study developed a biohybrid nanovesicle platform (DFO@HEVs) to address diabetic wound healing by combining EVs and NVs with DFO. This dual-targeting system leveraged CXCR4-mediated endothelial homing and ITGB2-dependent inflammation targeting to deliver DFO specifically to wound sites. DFO@HEVs could restore vascular regeneration through HIF-1α/VEGF activation, suppress ferroptosis via Nrf2/GPX4 signaling, and reprogram macrophages toward the anti-inflammatory M2 phenotype, effectively disrupting the oxidative-inflammatory-ferroptosis cycle. *In vivo*, DFO@HEVs could accelerate wound closure, reduce neutrophil infiltration, and enhance collagen remodeling, demonstrating their therapeutic potential. This modular platform, which integrated endothelial repair, antioxidant defense, and immunomodulation, offers a versatile strategy for diabetic wound management and paves the way for next-generation nanotherapeutics that target complex chronic diseases.

## Supplementary Material

tkag004_Supplemental_Files

## Data Availability

All data sources could be available to readers on request.
