# Supplementary material for: Hybrid nanovesicles promote diabetic wound healing via dual-targeted multimodal therapy
Source: Burns Trauma. 2026 Jan 11;14:tkag004. doi: 10.1093/burnst/tkag004 (PMC13103739; doi:10.1093/burnst/tkag004)
Supplement: tkag004_Supplemental_Files [file tkag004_supplemental_files.zip › Supplementary File. docx.docx]

Supporting Information for

**Hybrid Nanovesicles Promote Diabetic Wound Healing via Dual-Targeted Multimodal Therapy**

**Supporting Figures**

Figure S1.


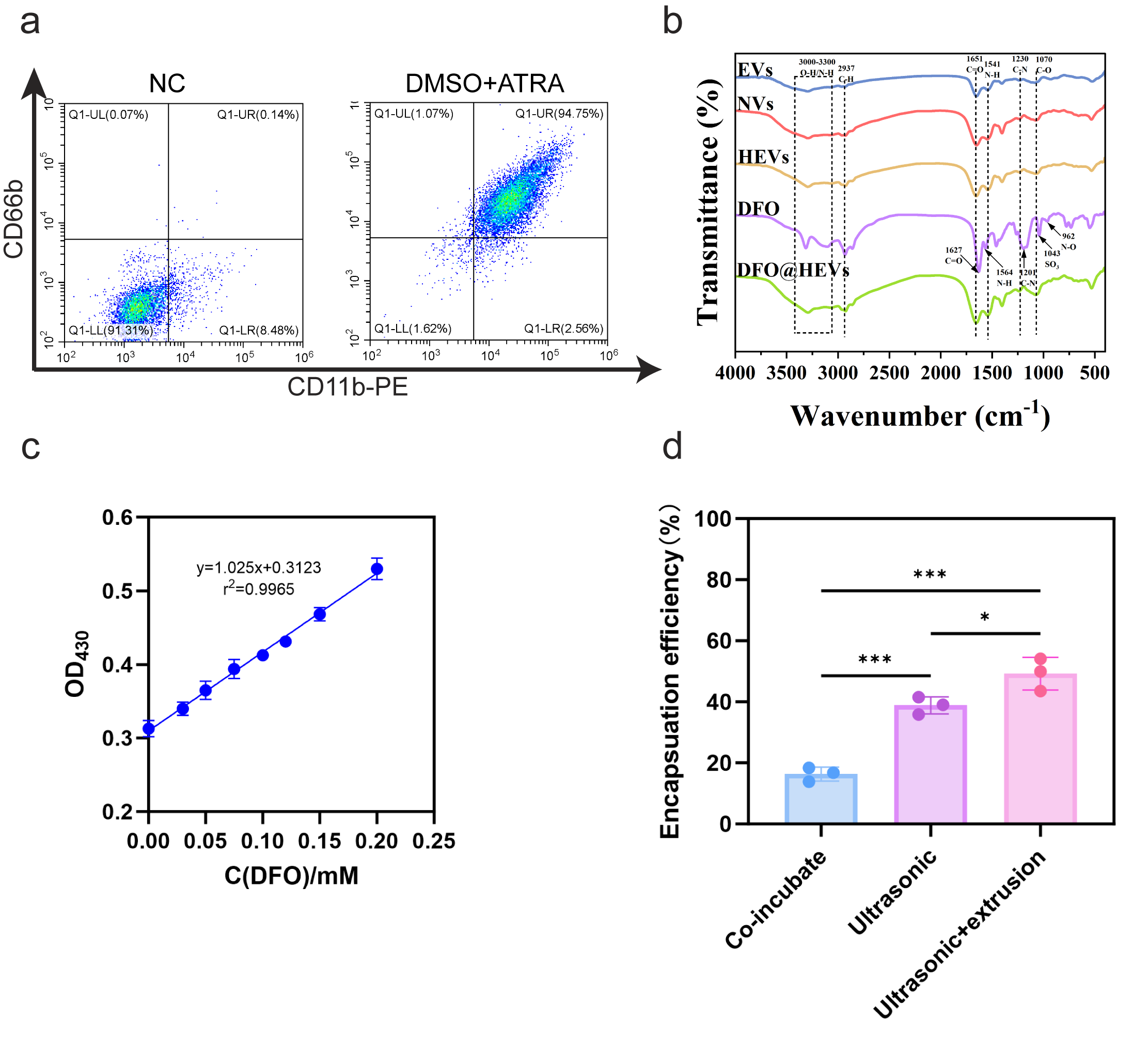


# Figure S1. ****Manufacture and Characterization of DFO@HEVs.**** (a) The expression of CD11b^+^/CD66b^+^ in HL-60 cells was induced by 1.3% DMSO and 1μM ATRA within 7 days (n = 3). (b) FTIR spectra of EVs, NVs, HEVs, DFO and DFO@HEVs. (c) The release curve of DFO@HEVs. (d)The encapsulation efficiency of DFO@HEVs manufactured by different methods. ***EVs* endothelial-derived extracellular vesicles, *NVs* neutrophil-derived extracellular vesicles, *HEVs* hybrid extracellular vesicles, *DFO@HEVs* DFO-loaded hybrid extracellular vesicles, *DFO* deferoxamine, *ATRA* Retinoic acid**

Figure S2.


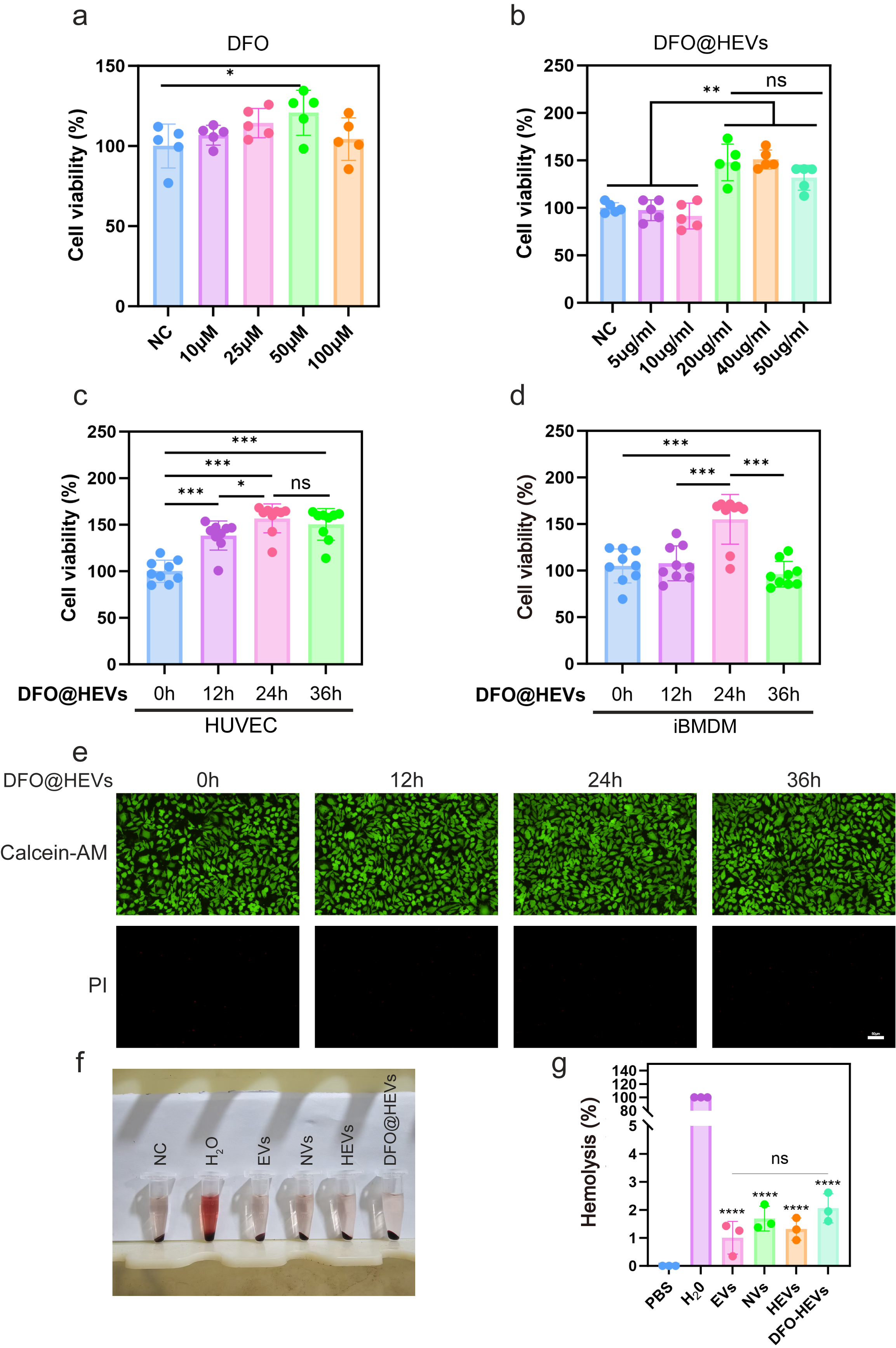


**Figure S2. Biocompatibility of DFO@HEVs.** (a, b) Cell viability of HUVEC after incubation with different concentrations of DFO and DFO@HEVs for 24 h (n=9). (c, d) Cell viability of HUVEC and iBMDMs after incubation with DFO@HEVs for different time. (n = 5). (e) Calcein-AM/PI staining of HUVEC incubated with DFO@HEVs for different time.(n = 5). Scale bar: 50μm. (f) Digital photographs of hemolysis for vesicles treatment. (g) Relative hemolysis percentage (n = 3). Data were displayed as mean ± SD. Data were assessed using one-way ANOVA and Tukey post hoc, **P* < 0.05, ***P* < 0.01, ****P* < 0.001, *****P* < 0.0001. ***EVs* endothelial-derived extracellular vesicles, *NVs* neutrophil-derived extracellular vesicles, *HEVs* hybrid extracellular vesicles, *DFO@HEVs* DFO-loaded hybrid extracellular vesicles, *DFO* deferoxamine**

Figure S3.

**
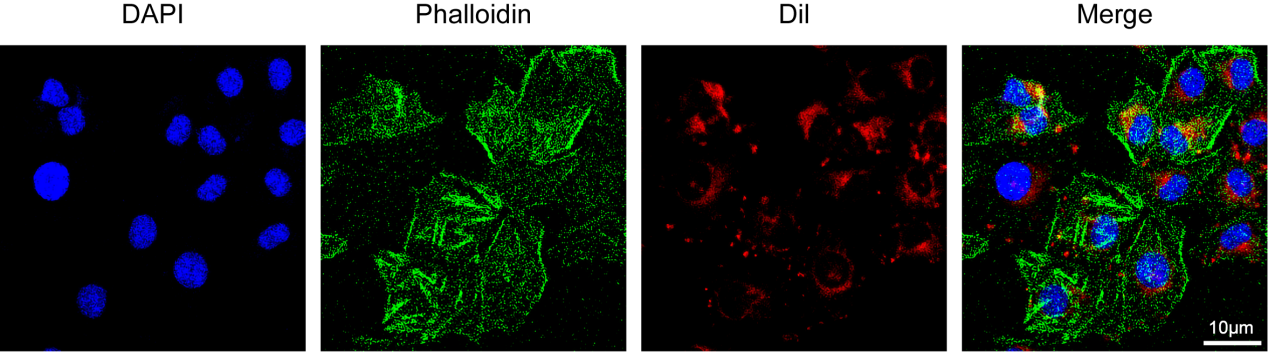
Figure S3. Internalization of DFO@HEVs in HUVECs.** CLSM images of DFO@HEVs uptake by HUVECs. Scale bar: 10μm. *DAPI* 4',6-Diamidino-2-phenylindole, *CLSM* Confocal Laser Scanning Microscopy, *Dil* 1,1'-dioctadecyl-3,3,3,3'-tetramethylindocarbocyanine perchlorate

Figure S4

**
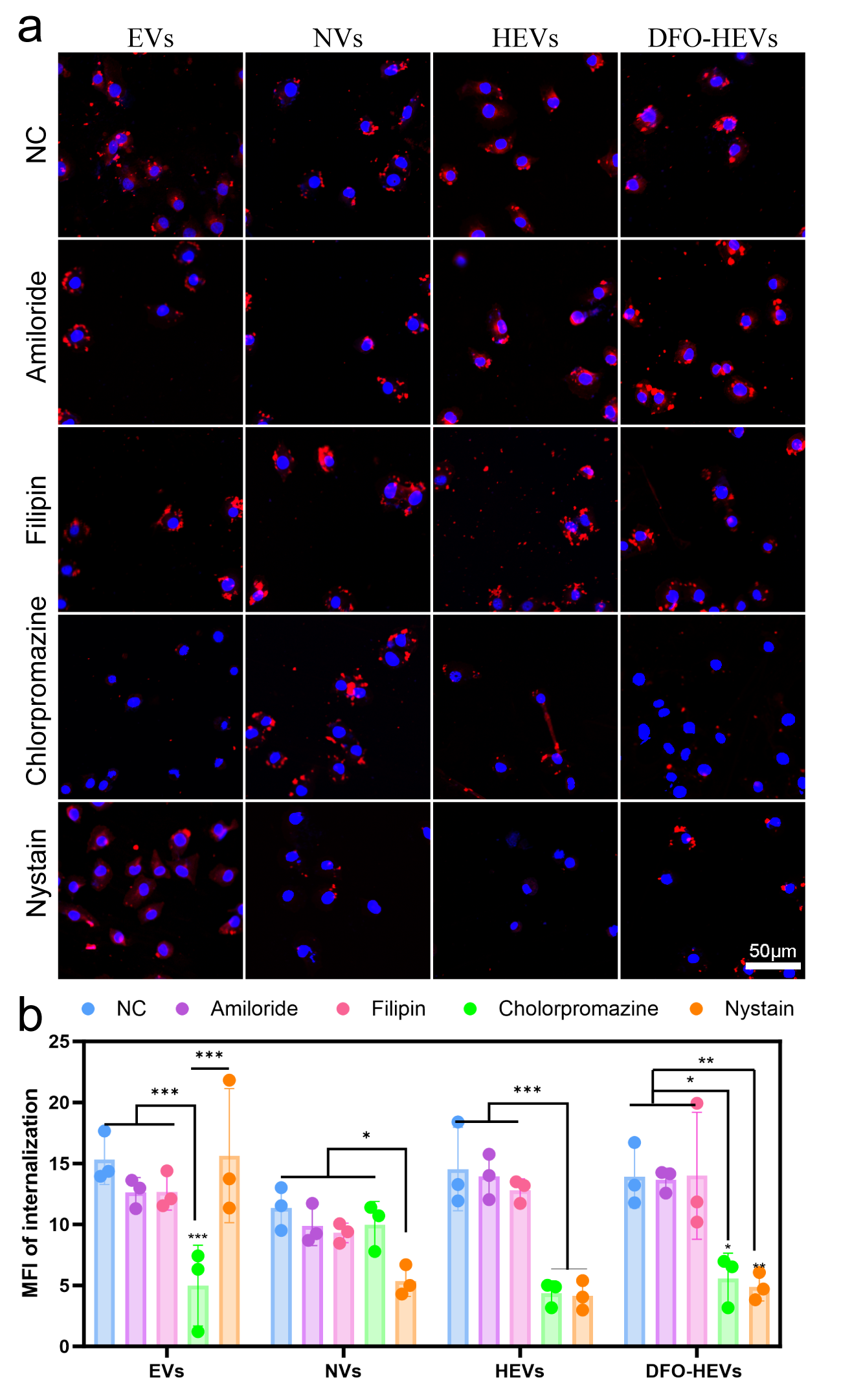
Figure S4. Internalization mechanism of different vesicles.** (a, b) CLSM image of internalization mechanism of different vesicles (n = 3) and corresponding statistical analysis of internalization ratio. Scale bar: 50μm. Data were displayed as mean ± SD. Data were assessed using one-way ANOVA and Tukey post hoc, **P* < 0.05, ***P* < 0.01, ****P* < 0.001, *****P* < 0.0001. ***EVs* endothelial-derived extracellular vesicles, *NVs* neutrophil-derived extracellular vesicles, *HEVs* hybrid extracellular vesicles, *DFO-HEVs* DFO-loaded hybrid extracellular vesicles**

Figure S5


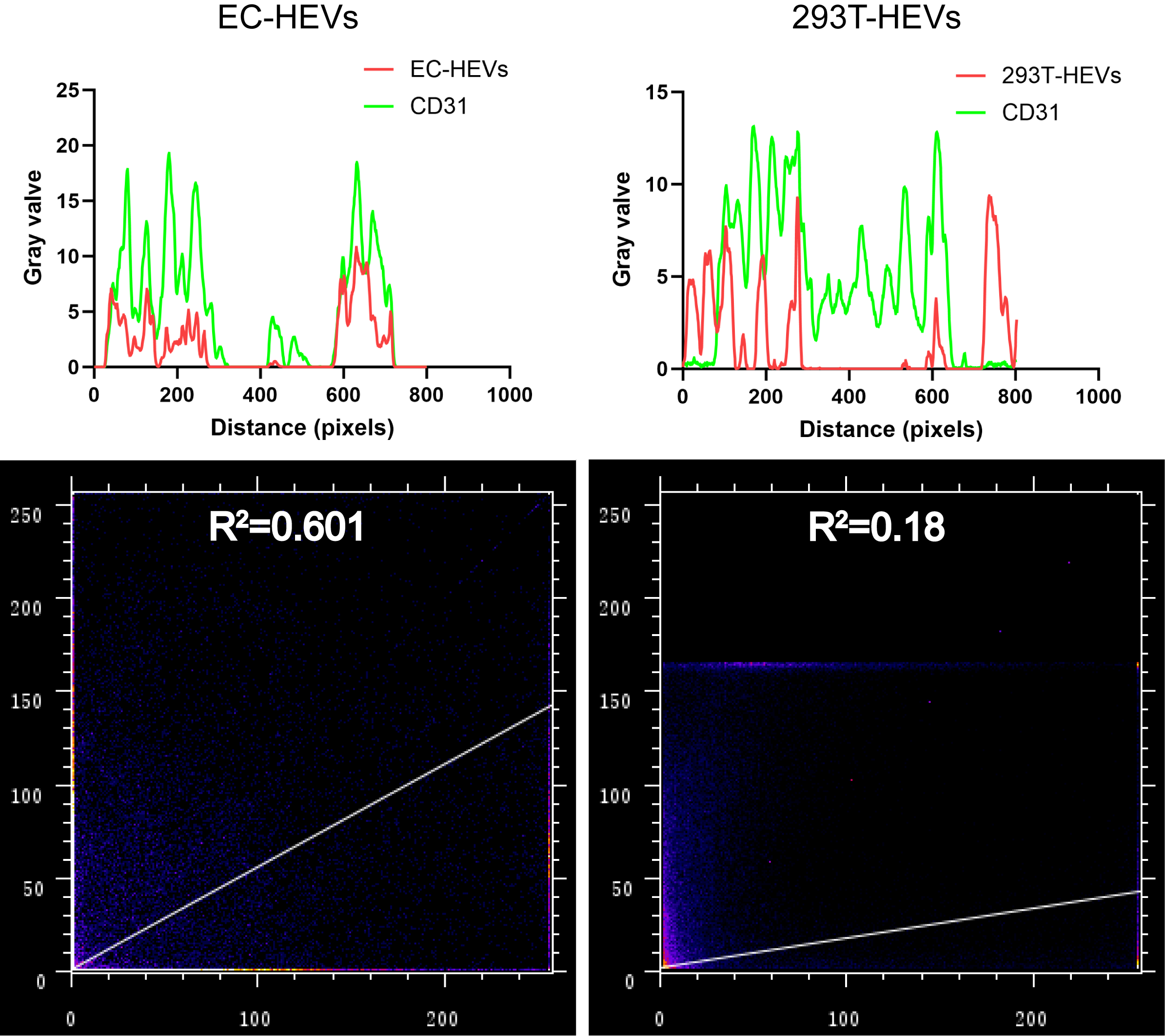


**Figure S5. Co-localization analysis of EC-HEVs and 293T-HEVs with CD31.** Corresponding fluorescence intensity trace in the merged image were plotted. Peak overlapping indicated the co-localization of HEVs and CD31.293T-HEV hybrid extracellular vesicles Hybrid extracellular vesicles derived from the fusion of 293T cell-derived and neutrophil-derived extracellular vesicles, EC-HEV hybrid extracellular vesicles Hybrid extracellular vesicles derived from the fusion of endothelial-derived and neutrophil-derived extracellular vesicles, CD31 platelet endothelial cell adhesion molecule-1

Figure S6


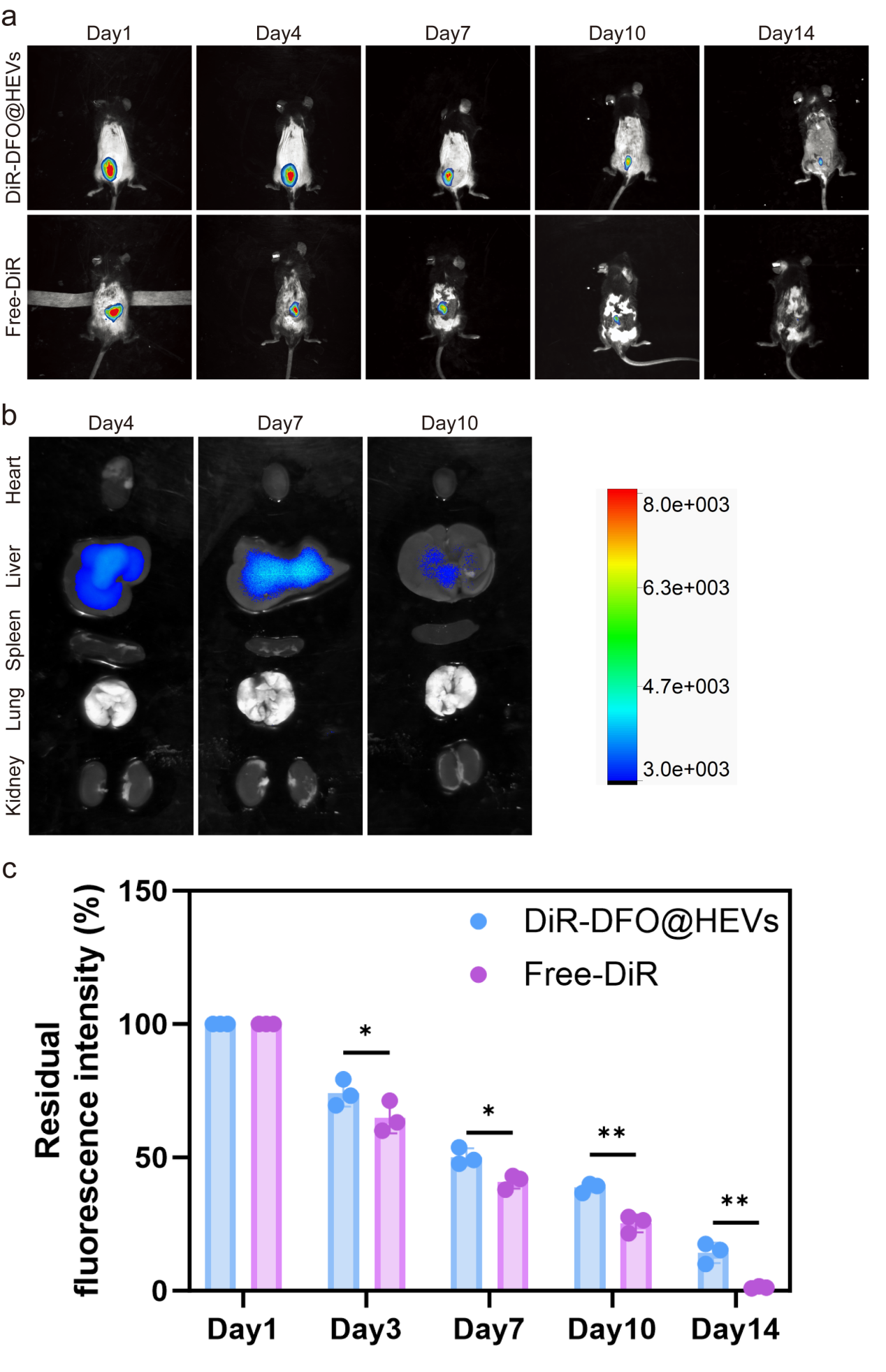


**Figure S6. ***In vivo* clearance and biodistribution of DFO@HEVs.**** (a) In vivo tracking of subcutaneously injected DiR-labeled DFO@HEVs. (b) In vivo imaging of major organs on days 3, 7, and 14. (c) Representative fluorescence imaging of mice wounds treated with 50 μg DiR-labeled DFO@HEVs or Free DiR was detected at indicated time points (n = 3). Data were displayed as mean ± SD. Data were assessed using one-way ANOVA and Tukey post hoc, **P* < 0.05, ***P* < 0.01, ****P* < 0.001, *****P* < 0.0001. ***DFO-HEVs* DFO-loaded hybrid extracellular vesicles,** *DiR* 1,1'-Dioctadecyl-3,3,3',3'-tetramethylindotricarbocyanine iodide

Figure S7


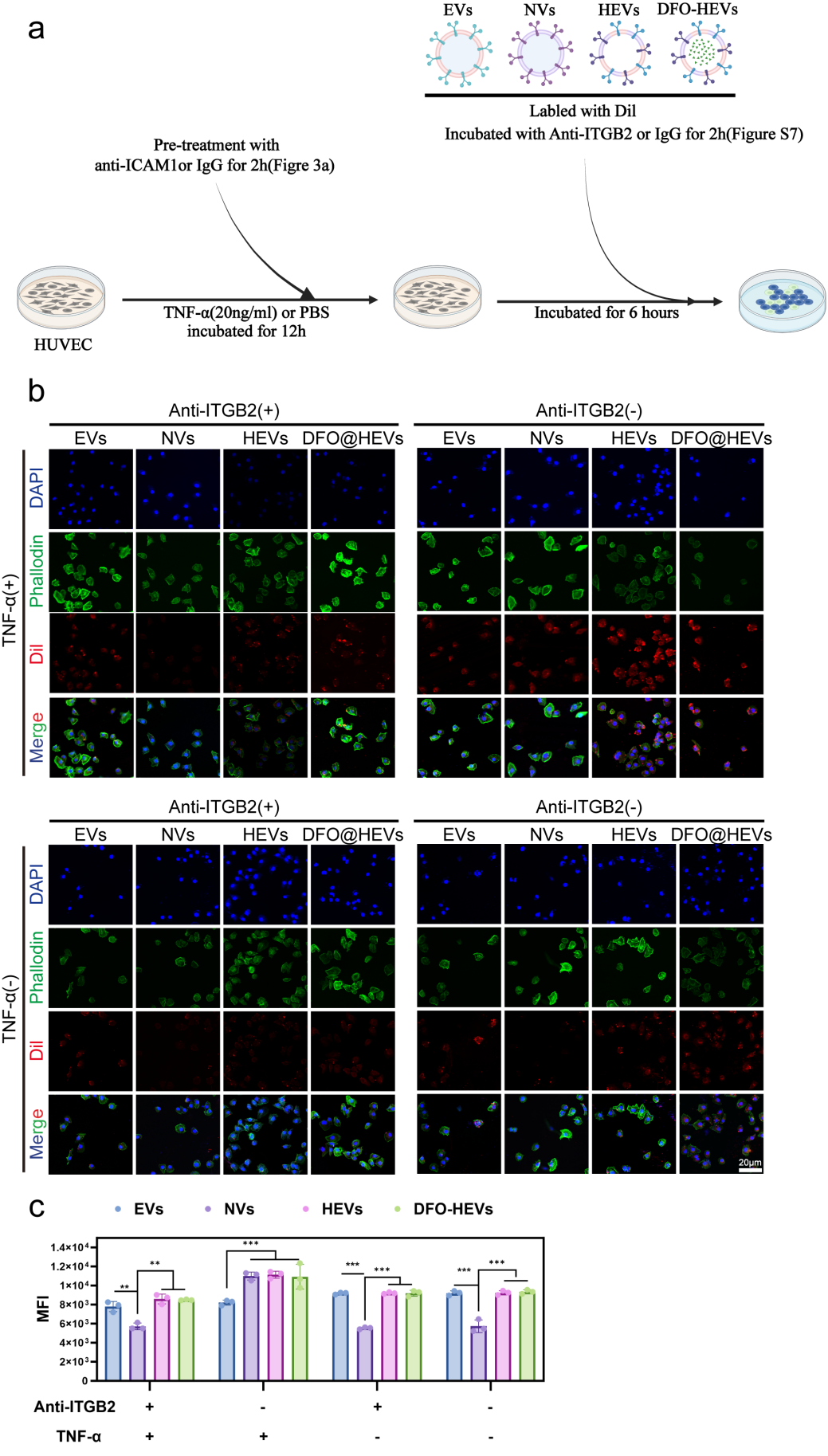


**Figure S7. **DFO@HEVs target inflamed HUVEC through ITGB2.**** (a) Scheme of Figure 2a and Figure S7b. (b, c) Fluorescence images of HUVEC uptake hybrid vesicles and neutrophil after hybrid vesicles pretreated with or without anti-β2 integrin antibody (n = 3). Scale bar: 20μm. Data were displayed as mean ± SD. Data were assessed using one-way ANOVA and Tukey post hoc, **P* < 0.05, ***P* < 0.01, ****P* < 0.001, *****P* < 0.0001. *Anti-ICAM1* Anti intercellular adhesion molecule 1 antibody, ***EVs* endothelial-derived extracellular vesicles, NVs neutrophil-derived extracellular vesicles, *HEVs* hybrid extracellular vesicles, *DFO-HEVs* DFO-loaded hybrid extracellular vesicles, *TNF-****α* tumor necrosis factor-alpha, *CFSE* Carboxyfluorescein diacetate succinimidyl ester, *Anti-ITGB2* Anti β_2_ integrin antibody, *DAPI* 4',6-Diamidino-2-phenylindole, CFSE carboxyfluorescein succinimidyl ester

Figure S8


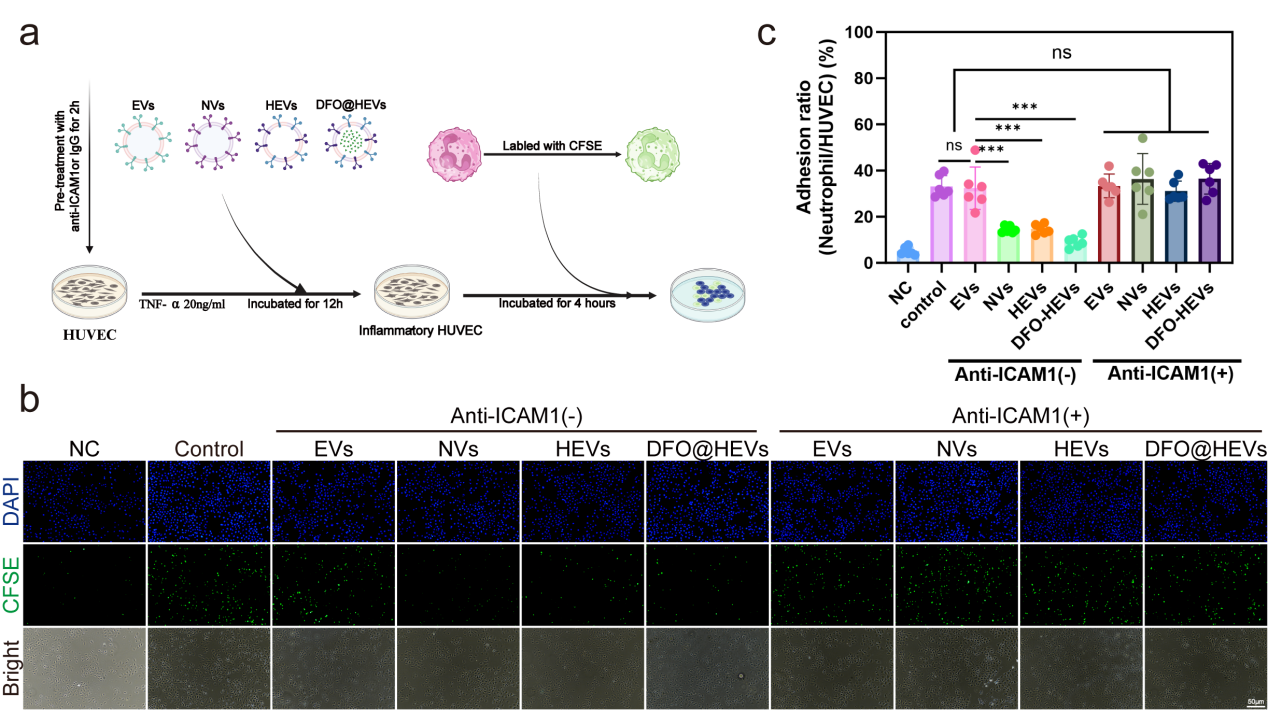


**Figure S8. DFO@HEVs reduce neutrophil adhesion by competitively binding to ICAM-1 on inflamed HUVEC.** (a)Scheme of Figure S8b. (b, c) Fluorescence images of neutrophil adhesion with HUVEC after hybrid vesicles pretreated with or without anti-β_2_integrin antibody (n = 6). Scale bar: 50μm. Data were displayed as mean ± SD. Data were assessed using one-way ANOVA and Tukey post hoc, **P* < 0.05, ***P* < 0.01, ****P* < 0.001, *****P* < 0.0001. *Anti-ICAM1* Anti intercellular adhesion molecule 1 antibody, ***EVs* endothelial-derived extracellular vesicles, *NVs* neutrophil-derived extracellular vesicles, *HEVs* hybrid extracellular vesicles, *DFO-HEVs* DFO-loaded hybrid extracellular vesicles, *TNF-****α* tumor necrosis factor-alpha, *CFSE* Carboxyfluorescein diacetate succinimidyl ester, *Anti-ITGB2* Anti β_2_ integrin antibody, *DAPI* 4',6-Diamidino-2-phenylindole, *CFSE* carboxyfluorescein succinimidyl ester

Figure S9


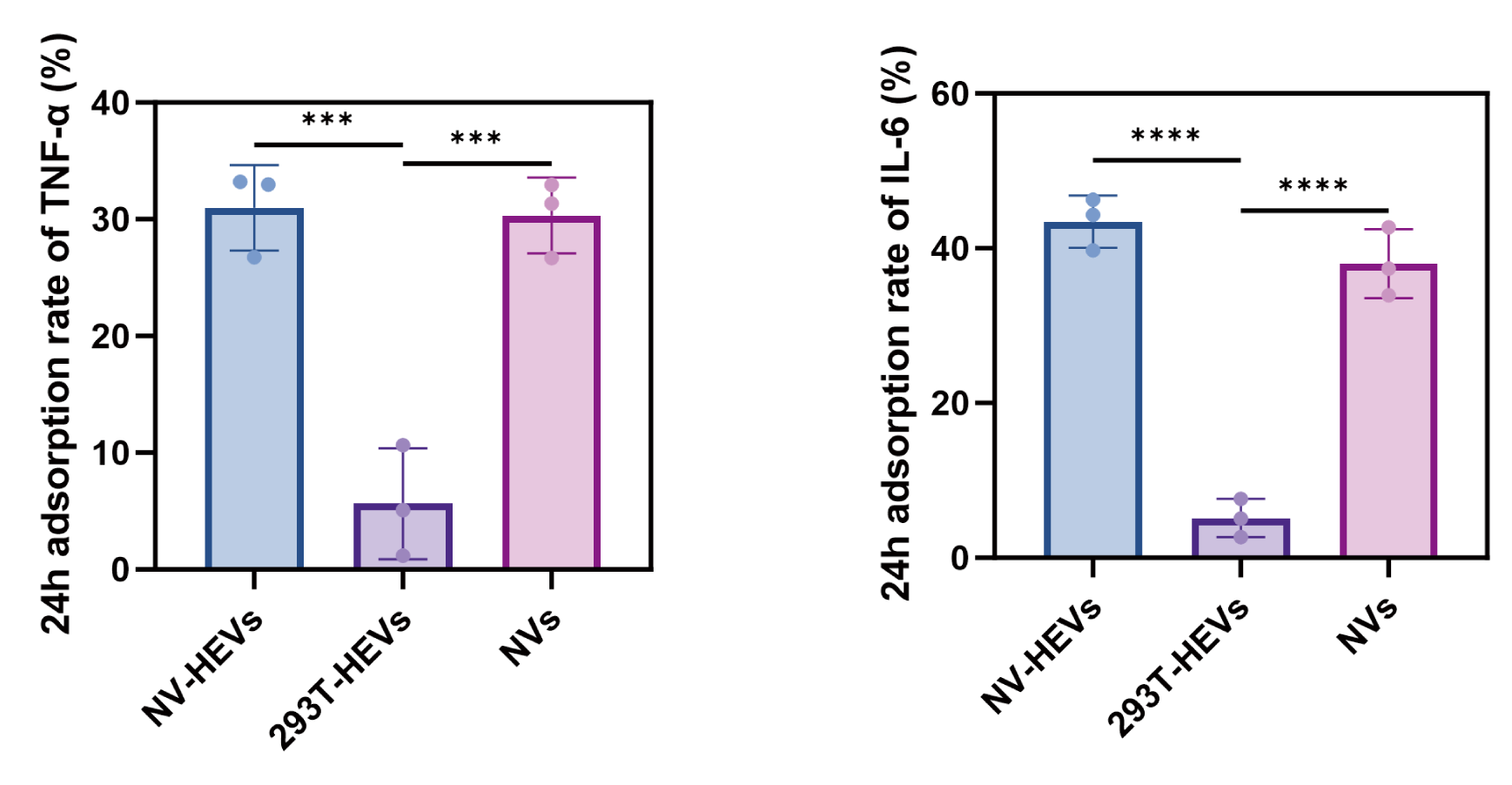


**Figure S9. Functional validation of DFO@HEVs in adsorbing inflammatory cytokines.** Adsorption ratio of different nanovesicles towards TNF-α and IL-6 at the initial cytokine concentration of 600 pg/mL (n = 3). Data were displayed as mean ± SD. Data were assessed using one-way ANOVA and Tukey post hoc, **P* < 0.05, ***P* < 0.01, ****P* < 0.001, *****P* < 0.0001. *TNF-α* tumor necrosis factor-α, *IL-6* interleukin- 6

Figure S10

**
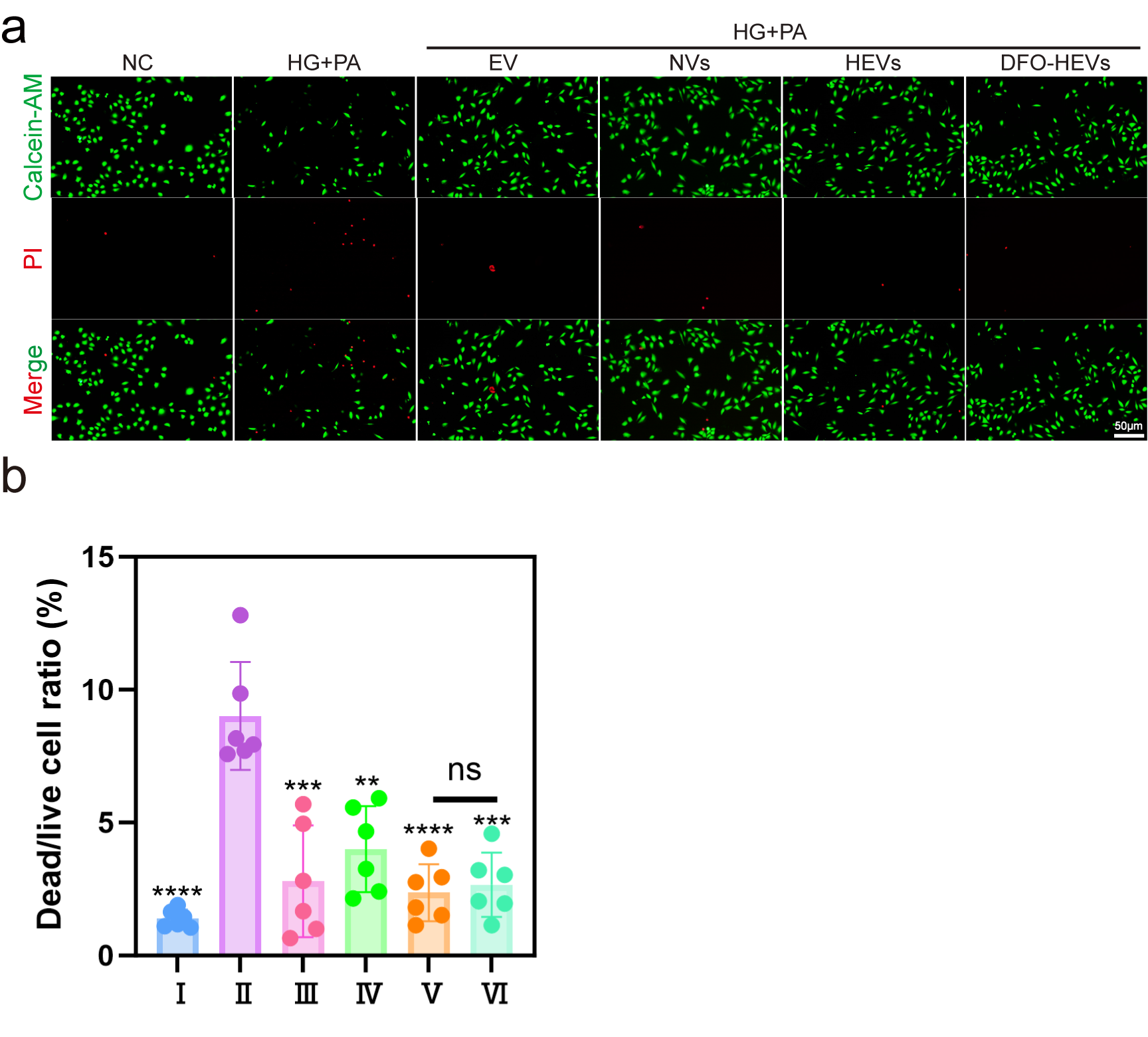
Figure S10. DFO@HEVs reduce HUVEC death induced by HG/PA.** (a) Representative calcein-AM/PI staining images of HUVECs and (b) corresponding statistical analysis of death to live cell rate (n = 6). Scale bar: 50μm. Data were displayed as mean±SD. Data were assessed using one-way ANOVA and Tukey post hoc, significance markers above bars (*, **, ***) indicate comparisons with Group II (asterisks: **P* < 0.05, ***P* < 0.01, ****P* < 0.001, *****P* < 0.0001). Additional horizontal brackets with corresponding symbols denote significant differences between other groups as indicated. ***EVs* endothelial-derived extracellular vesicles, *NVs* neutrophil-derived extracellular vesicles, *HEVs* hybrid extracellular vesicles, *DFO@HEVs* DFO-loaded hybrid extracellular vesicles, *HG/PA* high glucose and high palmitic acid**

Figure S11


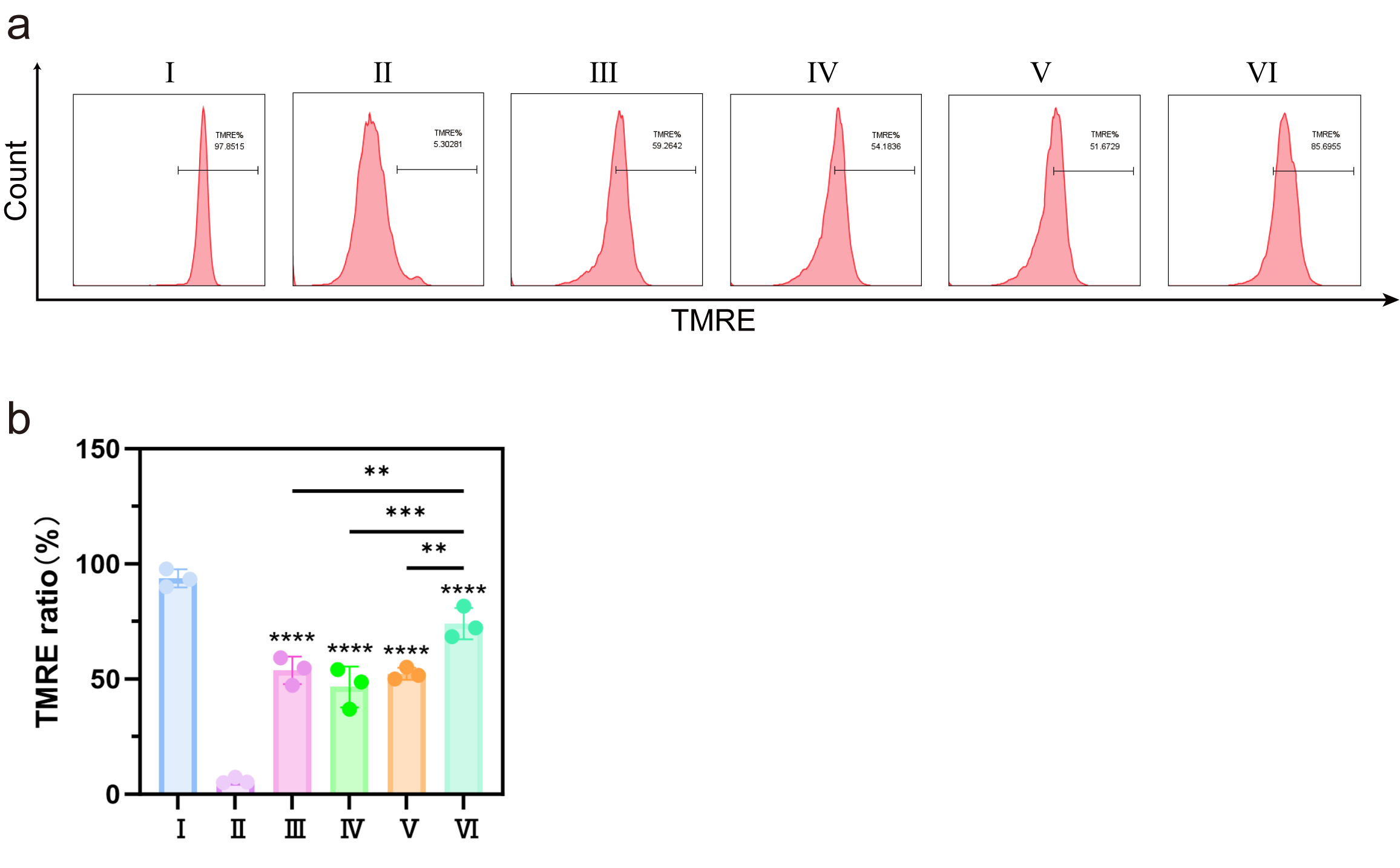


**Figure S11. DFO@HEVs restore mitochondrial membrane potential of HUVEC.** Representative FCM of TMRE staining and corresponding statistical analysis (n = 3). Data were displayed as mean ± SD. Data were assessed using one-way ANOVA and Tukey post hoc, significance markers above bars (*, **, ***) indicate comparisons with Group II (asterisks: **P* < 0.05, ***P* < 0.01, ****P* < 0.001, **** *P* < 0.0001). Additional horizontal brackets with corresponding symbols denote significant differences between other groups as indicated

Figure S12


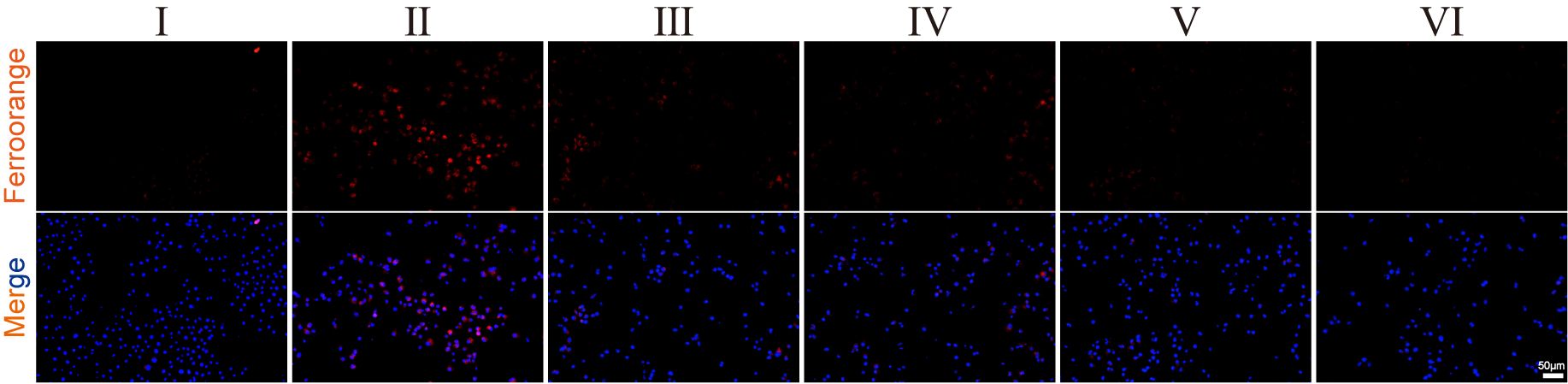


**Figure S12. DFO@HEVs reduce intracellular free Fe²⁺ in HUVEC.** Representative FerroOrange fluorescence images of endothelial cells after treatment with different extracellular vesicles. Scale bar:50μm

Figure S13


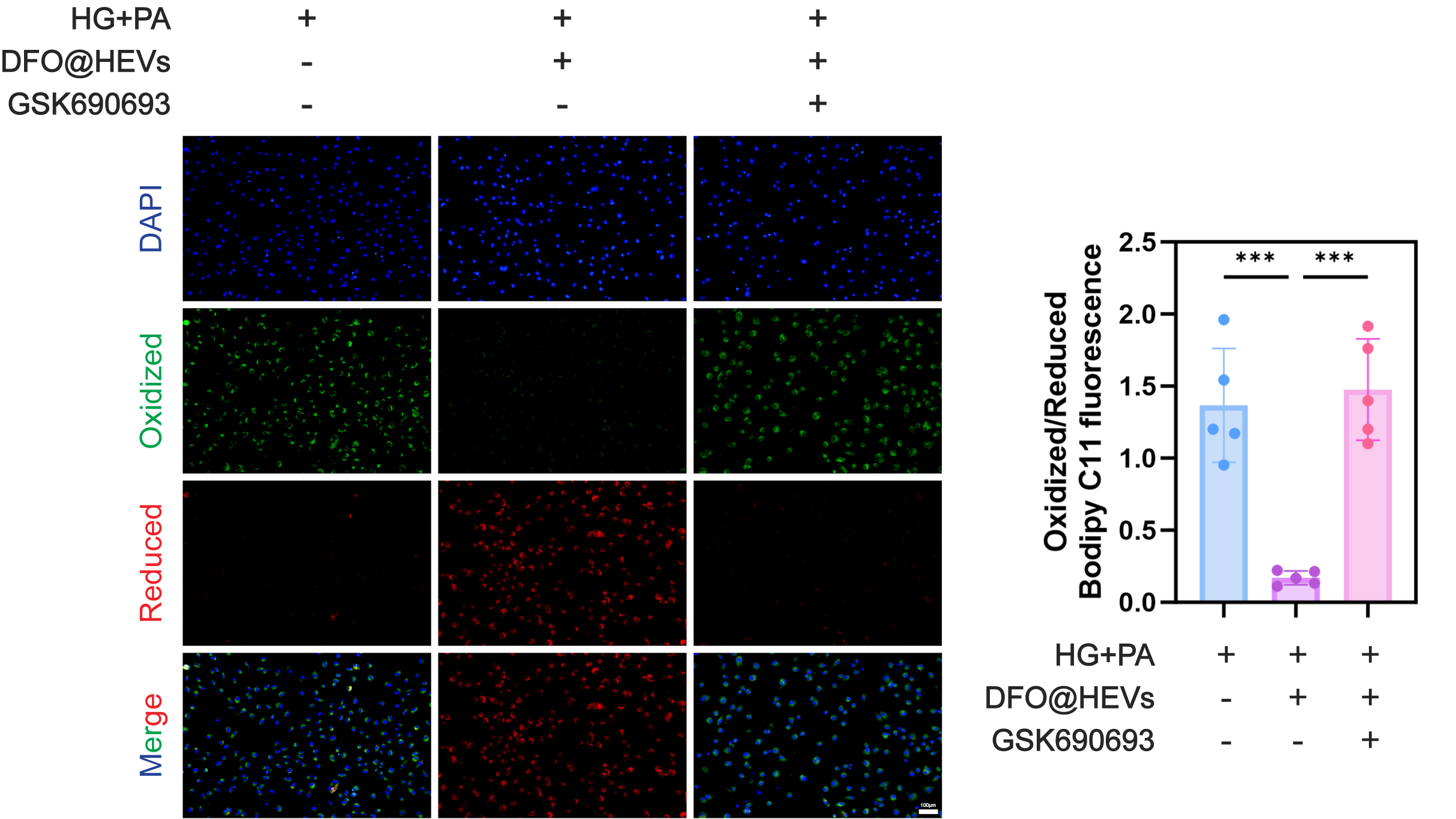


**Figure S13. AKT inhibitor reversed the protective effect of DFO@HEVs on HUVECs.** Representative Bodipy C11 fluorescence images of endothelial cells after be treated or not treated with GSK690693 (n = 5). Scale bar:100μm. Data were displayed as mean ± SD. Data were assessed using one-way ANOVA and Tukey post hoc, **P* < 0.05, ***P* < 0.01, ****P* < 0.001, *****P* < 0.0001. ***DFO@HEVs* DFO-loaded hybrid extracellular vesicles, *HG/PA* high glucose and high palmitic acid, *GSK690693*** ATP-competitive pan-Akt inhibitor

Figure S14


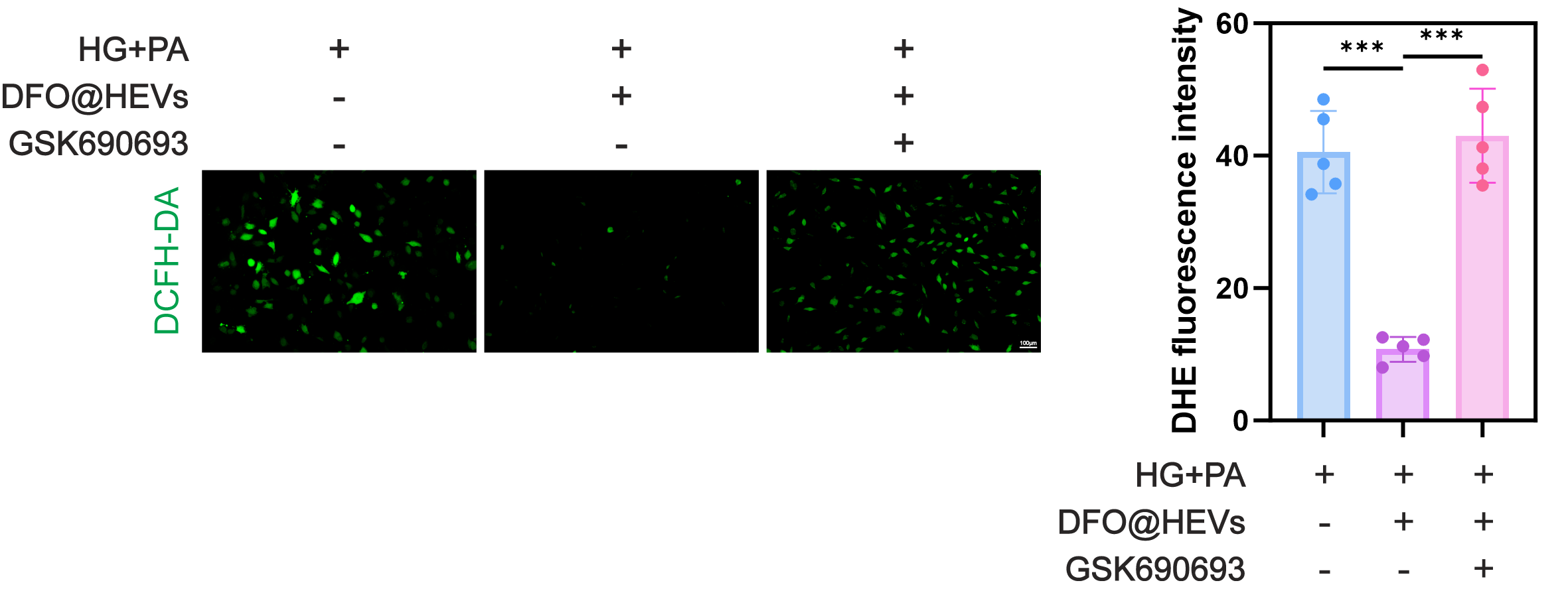


**Figure S14. AKT inhibitor reversed the protective effect of DFO@HEVs on HUVECs.** Representative DCFH-DA fluorescence images of endothelial cells after be treated or not treated with GSK690693 (n = 5). Scale bar:100μm. Data were displayed as mean ± SD. Data were assessed using one-way ANOVA and Tukey post hoc, **P* < 0.05, ***P* < 0.01, ****P* < 0.001, *****P* < 0.0001. ***DFO@HEVs* DFO-loaded hybrid extracellular vesicles, *HG/PA* high glucose and high palmitic acid, *GSK690693*** ATP-competitive pan-Akt inhibitor

Figure S15


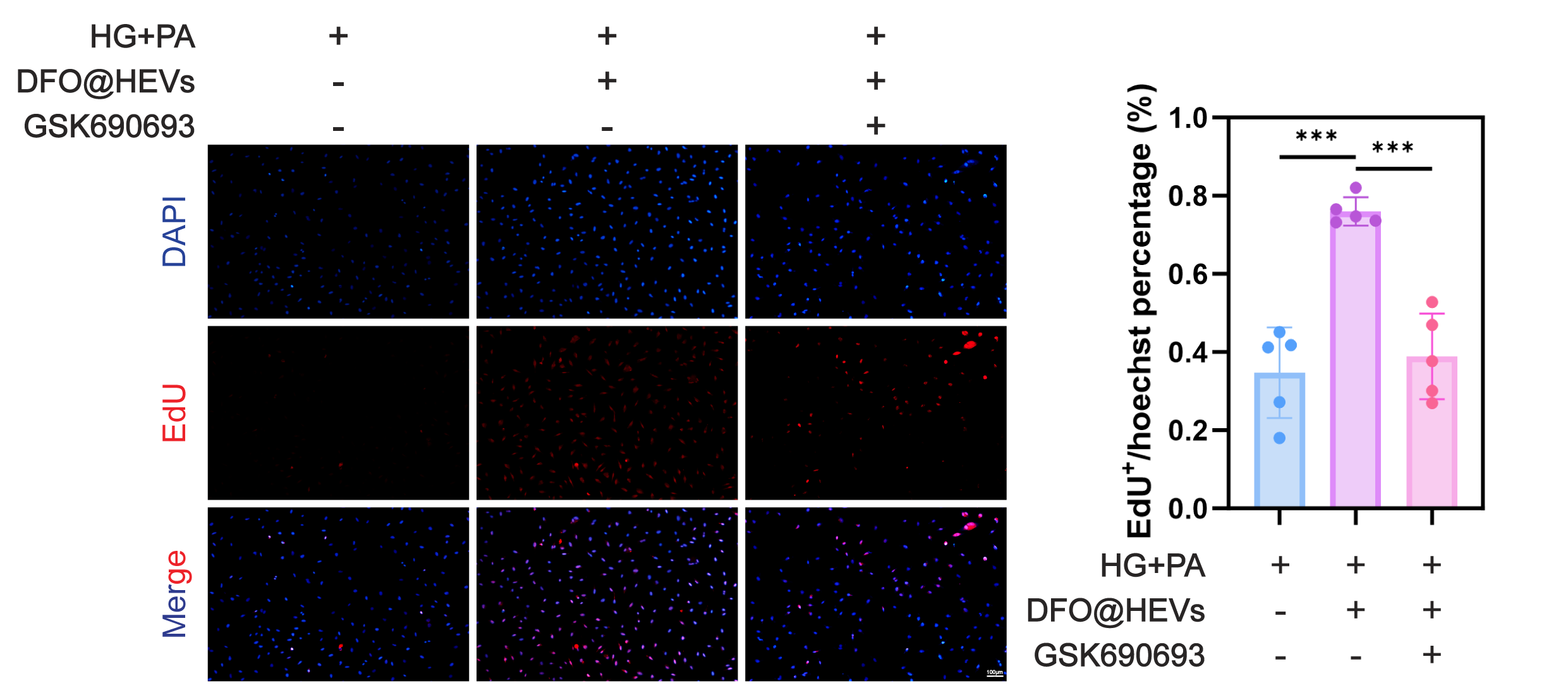


**Figure S15. AKT inhibitor reversed the protective effect of DFO@HEVs on HUVECs.** Representative EdU fluorescence images of endothelial cells after be treated or not treated with GSK690693 (n = 5). Scale bar:100μm. Data were displayed as mean ± SD. Data were assessed using one-way ANOVA and Tukey post hoc, **P* < 0.05, ***P* < 0.01, ****P* < 0.001, *****P* < 0.0001. ***DFO@HEVs* DFO-loaded hybrid extracellular vesicles, *HG/PA* high glucose and high palmitic acid, *GSK690693*** ATP-competitive pan-Akt inhibitor

Figure S16


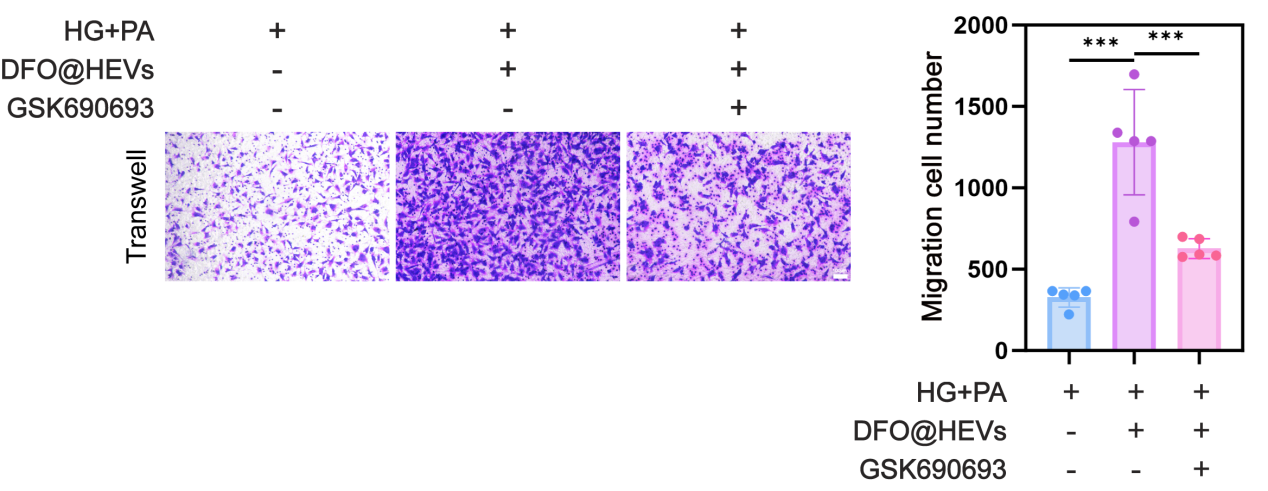


**Figure S16. AKT inhibitor reversed the protective effect of DFO@HEVs on HUVECs.** Representative transwell images of endothelial cells after be treated or not treated with GSK690693 (n = 5). Scale bar:100μm. Data were displayed as mean ± SD. Data were assessed using one-way ANOVA and Tukey post hoc, **P* < 0.05, ***P* < 0.01, ****P* < 0.001, *****P* < 0.0001. ***DFO@HEVs* DFO-loaded hybrid extracellular vesicles, *HG/PA* high glucose and high palmitic acid, *GSK690693*** ATP-competitive pan-Akt inhibitor

Figure S17


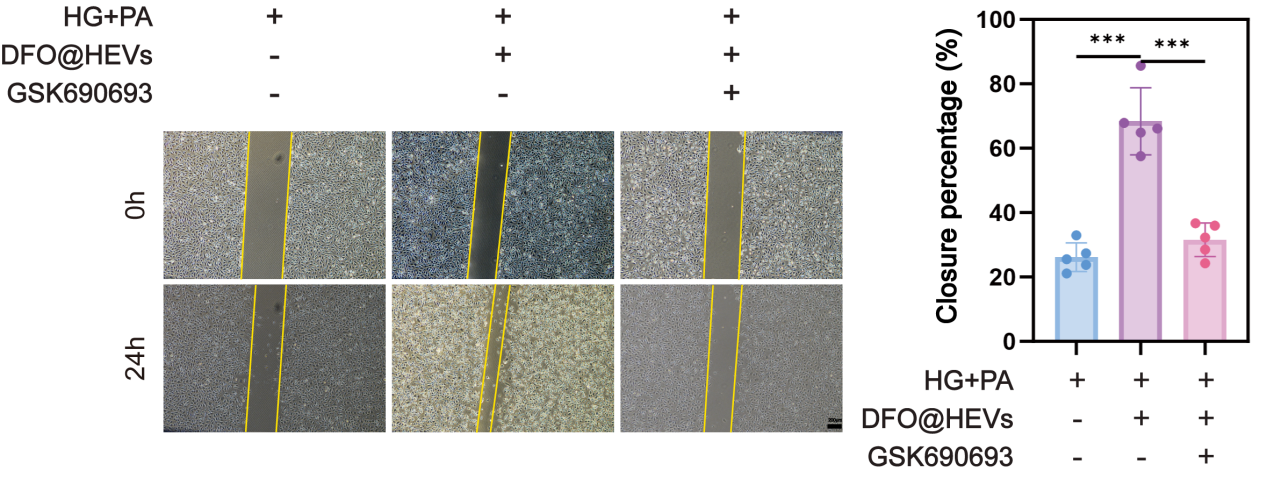


**Figure S17. AKT inhibitor reversed the protective effect of DFO@HEVs on HUVECs.** Representative scratch test images of endothelial cells after be treated or not treated with GSK690693 (n = 5). Scale bar:200μm. Data were displayed as mean ± SD. Data were assessed using one-way ANOVA and Tukey post hoc, **P* < 0.05, ***P* < 0.01, ****P* < 0.001, *****P* < 0.0001. ***DFO@HEVs* DFO-loaded hybrid extracellular vesicles, *HG/PA* high glucose and high palmitic acid, *GSK690693*** ATP-competitive pan-Akt inhibitor

Figure S18


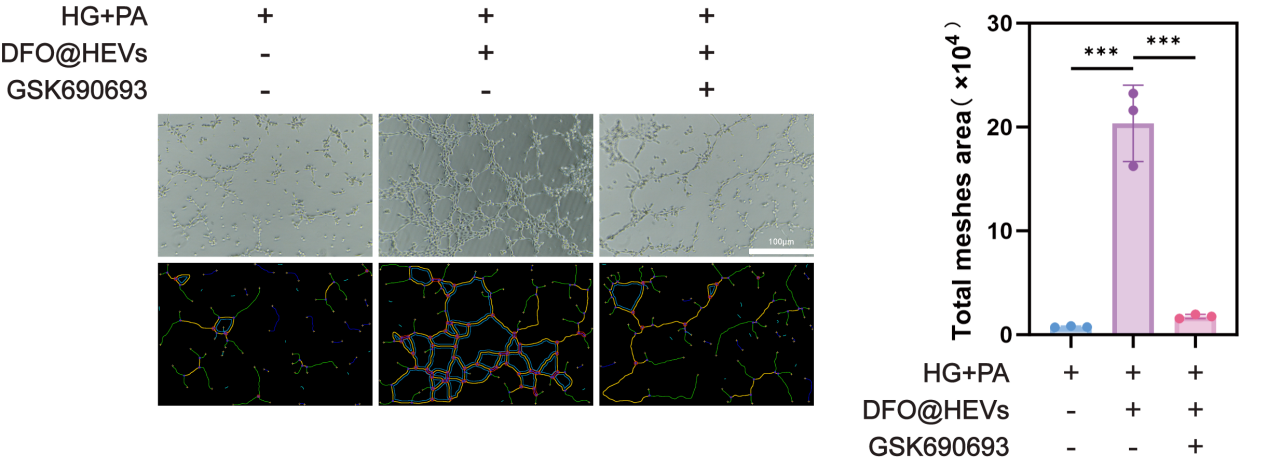


**Figure S18. AKT inhibitor reversed the protective effect of DFO@HEVs on HUVECs.** Representative tube formation images of endothelial cells after be treated or not treated with GSK690693 (n = 3). Scale bar:100μm. Data were displayed as mean ± SD. Data were assessed using one-way ANOVA and Tukey post hoc, **P* < 0.05, ***P* < 0.01, ****P* < 0.001, *****P* < .0001. ***DFO@HEVs* DFO-loaded hybrid extracellular vesicles, *HG/PA* high glucose and high palmitic acid, *GSK690693*** ATP-competitive pan-Akt inhibitor

Figure S19


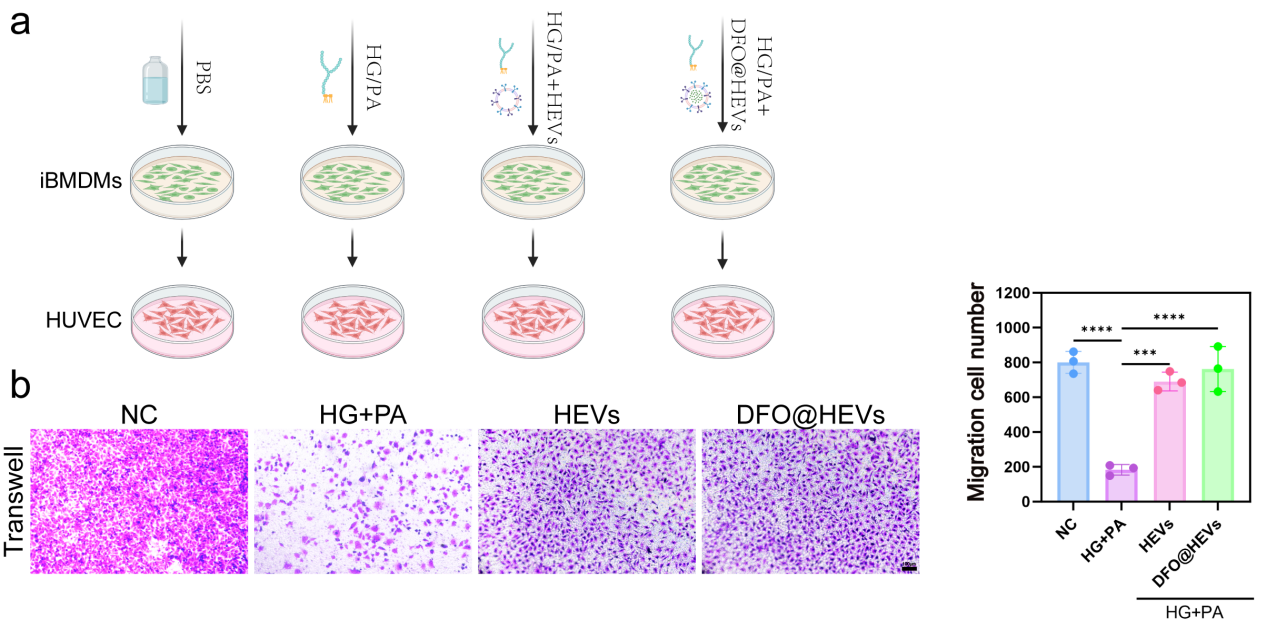


**Figure S19. DFO@HEVs restore endothelial cell function by improving the immune microenvironment.** (a) Scheme of test in Figure S19b and Figure S20.(b) Representative transwell images of endothelial cells after be treated with Macrophage supernatant after different treatment (n = 3). Scale bar:100μm. Data were displayed as mean ± SD. Data were assessed using one-way ANOVA and Tukey post hoc, **P* < 0.05, ***P* < 0.01, ****P* < 0.001, *****P* < 0.0001. *PBS* phosphate buffer saline, ***HEVs* hybrid extracellular vesicles, *DFO@HEVs* DFO-loaded hybrid extracellular vesicles, *HG/PA* high glucose and high palmitic acid, *GSK690693*** ATP-competitive pan-Akt inhibitor

Figure S20


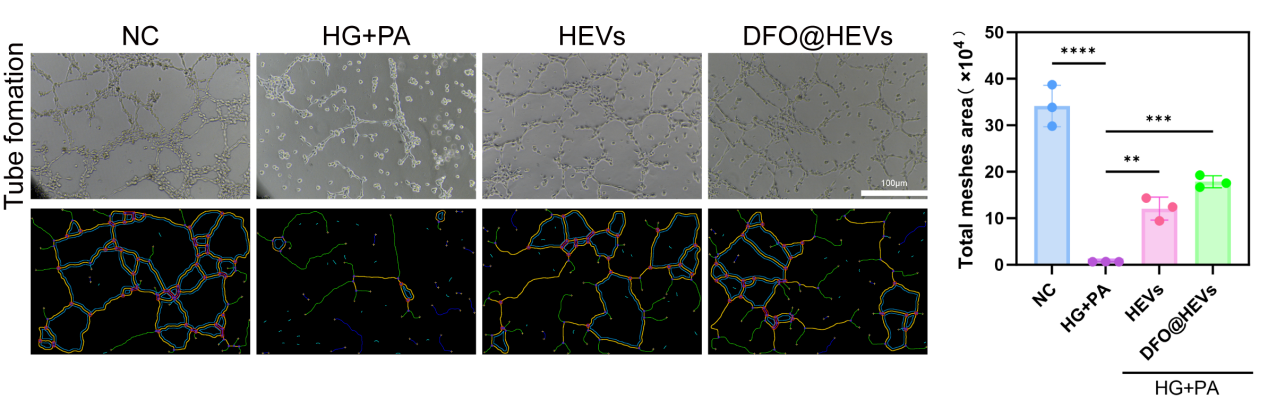


**Figure S20. DFO@HEVs restore endothelial cell function by improving the immune microenvironment.** Representative tube formation images of endothelial cells after be treated with Macrophage supernatant after different treatment (n = 3). Scale bar:100μm. Data were displayed as mean ± SD. Data were assessed using one-way ANOVA and Tukey post hoc, **P* < 0.05, ***P* < 0.01, ****P* < 0.001, *****P* < 0.0001. *PBS* phosphate buffer saline, ***HEVs* hybrid extracellular vesicles, *DFO@HEVs* DFO-loaded hybrid extracellular vesicles, *HG/PA* high glucose and high palmitic acid, *GSK690693*** ATP-competitive pan-Akt inhibitor

Figure S21


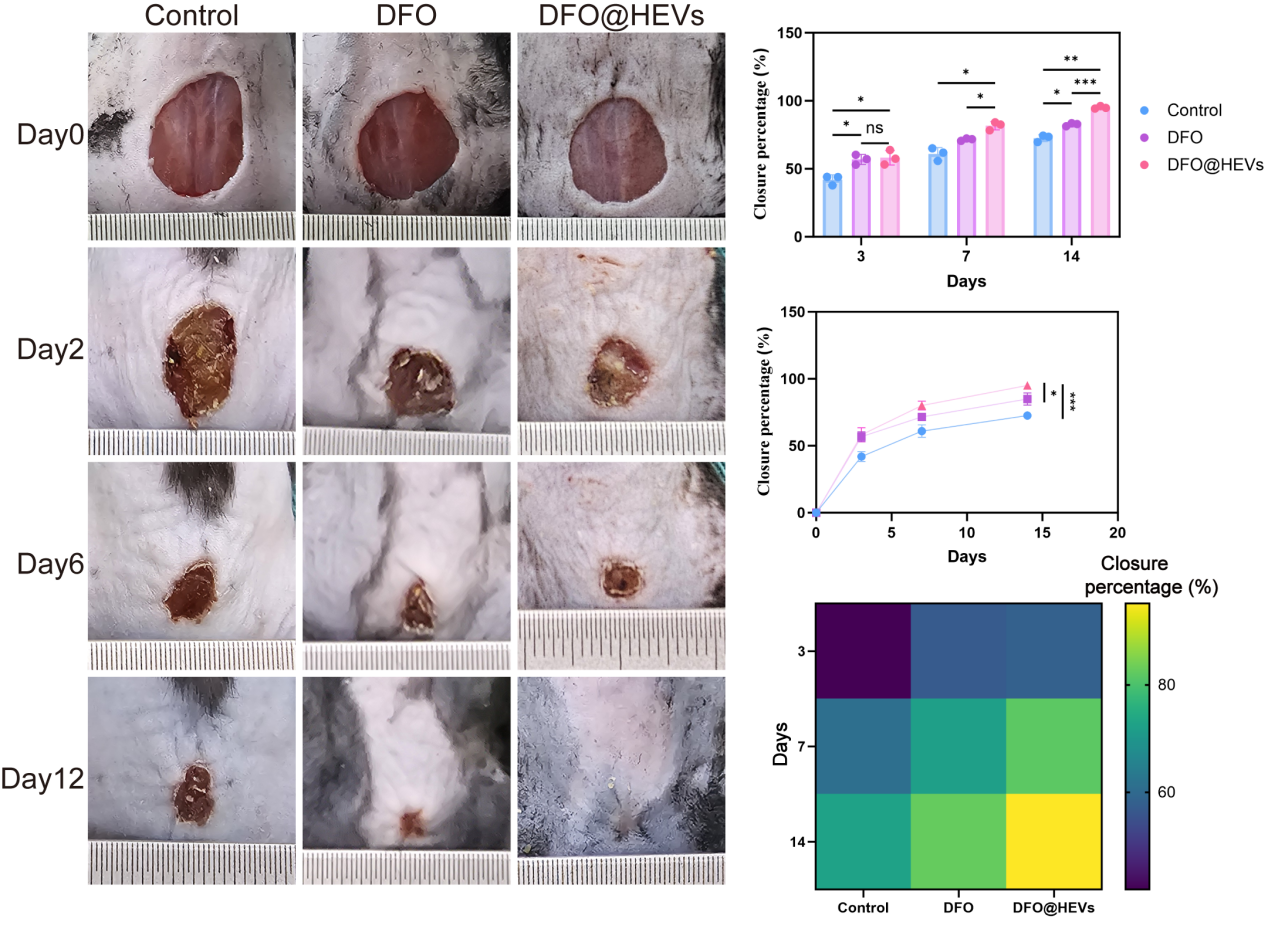


**Figure S21. DFO@HEVs promoted wound healing better than free DFO *in vivo*.** Grouping information and representative images,monitoring and statistics of wound closure at days 0, 2, 6, and 12 (n = 3). Data were displayed as mean ± SD. Data were assessed using one-way ANOVA and Tukey post hoc, **P* < 0.05, ***P* < 0.01, ****P* < 0.001, *****P* < 0.0001. ***DFO@HEVs* DFO-loaded hybrid extracellular vesicles,** *DFO* deferoxamine

Figure S22


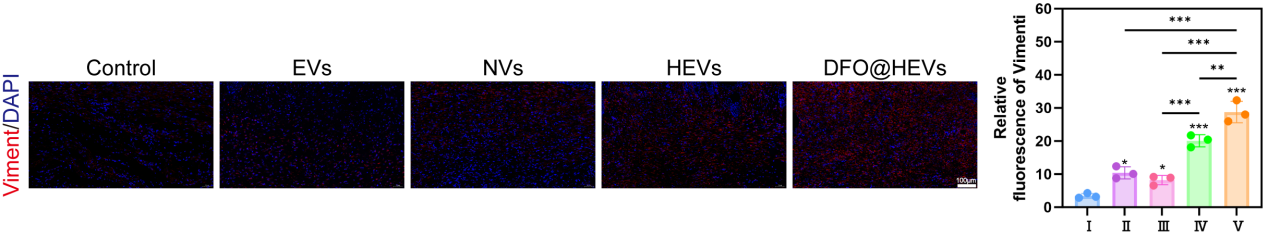


**Figure S22. DFO@HEVs enhanced fibroblast activity in the wound area.** Representative images of immunofluorescence staining of Vimenti (n = 3). Scale bar: 100μm. Data were displayed as mean ± SD. Data were assessed using one-way ANOVA and Tukey post hoc, **P* < 0.05, ***P* < 0.01, ****P* < 0.001, *****P* < 0.0001. ***EVs* endothelial-derived extracellular vesicles, *NVs* neutrophil-derived extracellular vesicles, *HEVs* hybrid extracellular vesicles, *DFO@HEVs* DFO-loaded hybrid extracellular vesicles**

Figure S23


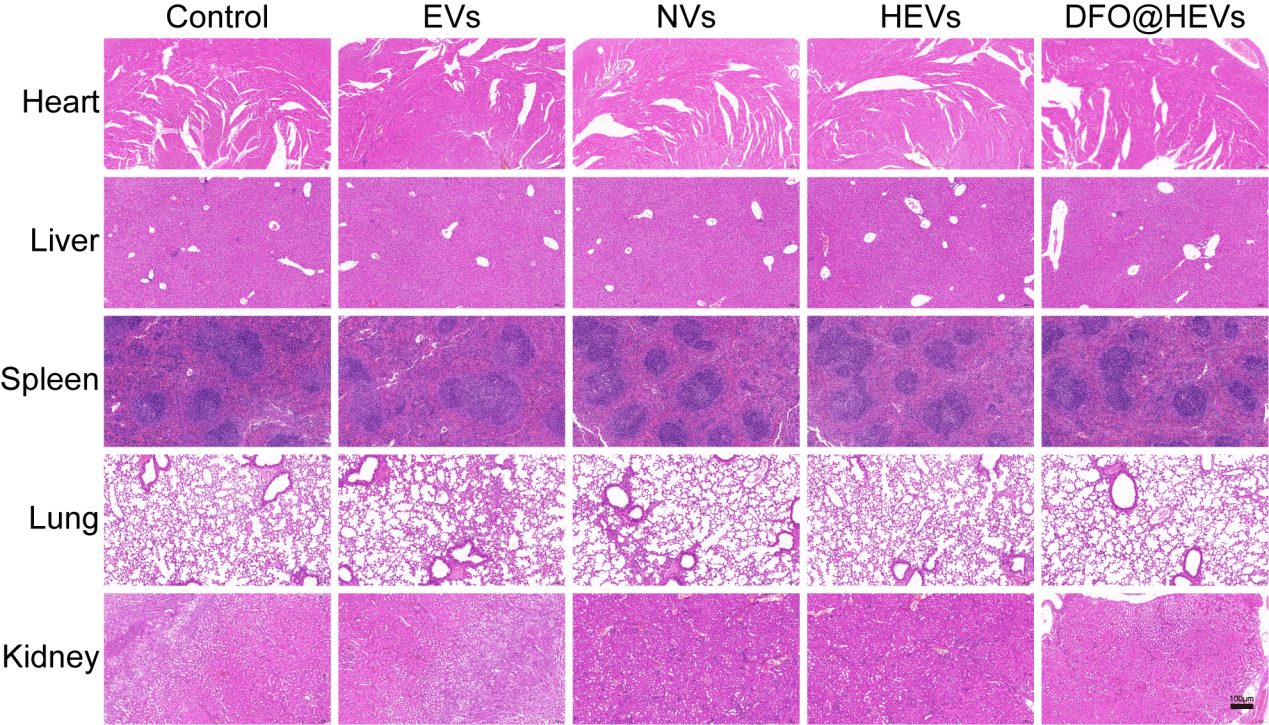


**Figure S23. Biocompatibility of DFO@HEVs *in vivo*.** H&E staining of the main organs (heart, liver, spleen, lung, and kidney) from diabetic mice with wounds in each group on day 14 post-treatment. Scale bar: 200μm. ***EVs* endothelial-derived extracellular vesicles, *NVs* neutrophil-derived extracellular vesicles, *HEVs* hybrid extracellular vesicles, *DFO@HEVs* DFO-loaded hybrid extracellular vesicles**

Figure S24


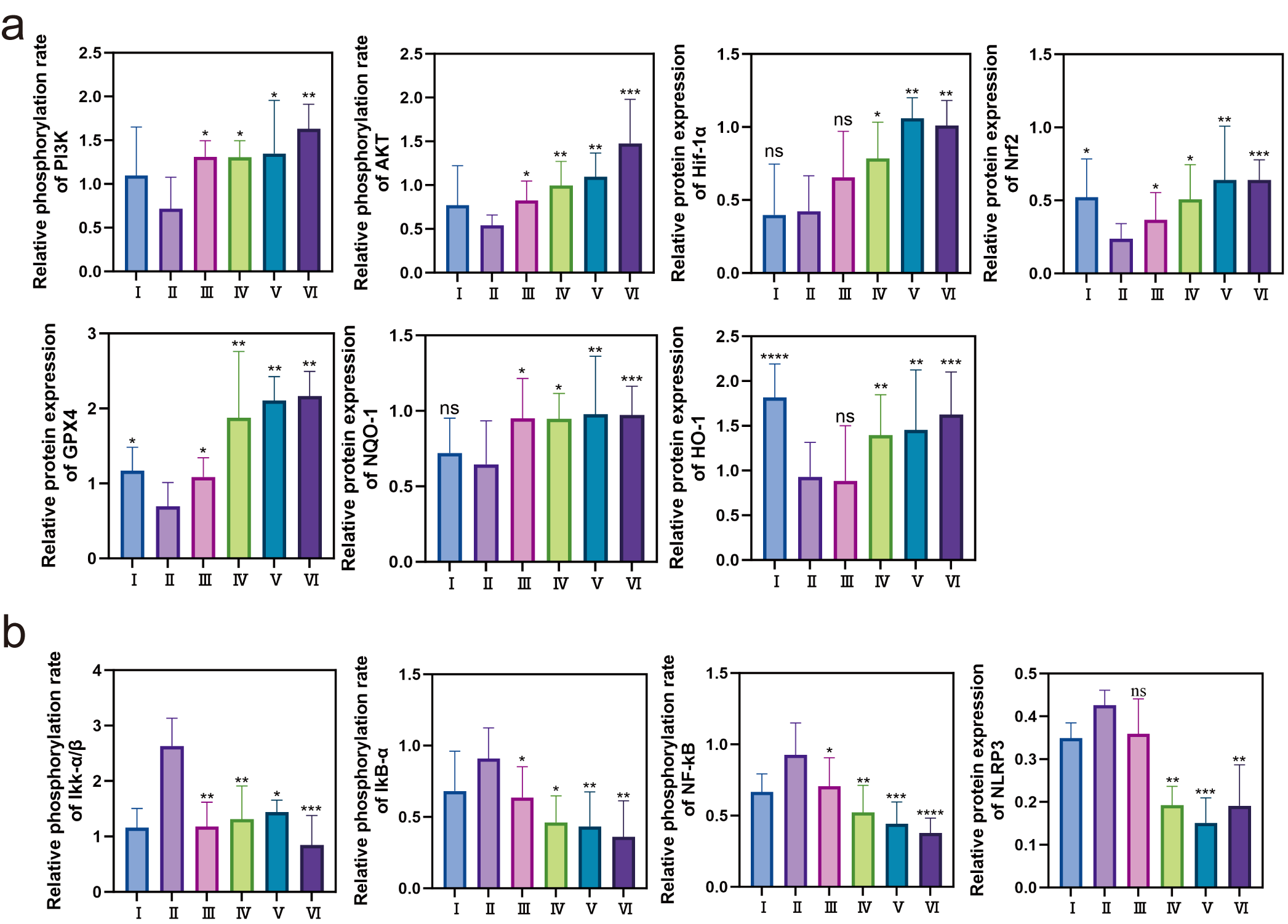


**Figure S24. Corresponding statistical analysis of western blot in figure 7, 8.** Data were displayed as mean ±SD. Data were assessed using one-way ANOVA and Tukey post hoc, significance markers above bars (*, **, ***) indicate comparisons with Group II (asterisks: **P* < 0.05, ***P* < 0.01, ****P* < 0.001, *****P* < 0.0001). Additional horizontal brackets with corresponding symbols denote significant differences between other groups as indicated. *HIF-1* α hypoxia inducible factor 1 subunit alpha, *PI3K* phosphatidylinositol 3-kinase, *P-PI3K* phosphorylated phosphatidylinositol 3-kinase, *AKT* protein kinase b, *P-AKT* phosphorylated protein kinase b, *Nrf2* nuclear factor erythroid 2-related factor 2, *NQO1* nad(p)h quinone oxidoreductase, *HO-1* heme oxygenase 1, *GPX4* glutathione peroxidase 4, *ACSL4* acyl-coa synthetase long chain family member 4, *iNOS* inducible nitric oxide synthase, *Arg-1* arginase-1, *IL* interleukin-10, *TNF-α* tumor necrosis factor-alpha, *Iκk* inhibitor of nuclear factor kappa-b kinase, *IκB* inhibitor of nuclear factor kappa-b, *NF-κB* nuclear factor kappa-b, *NLRP3* nod-like receptor family pyrin domain containing 3

Figure S25


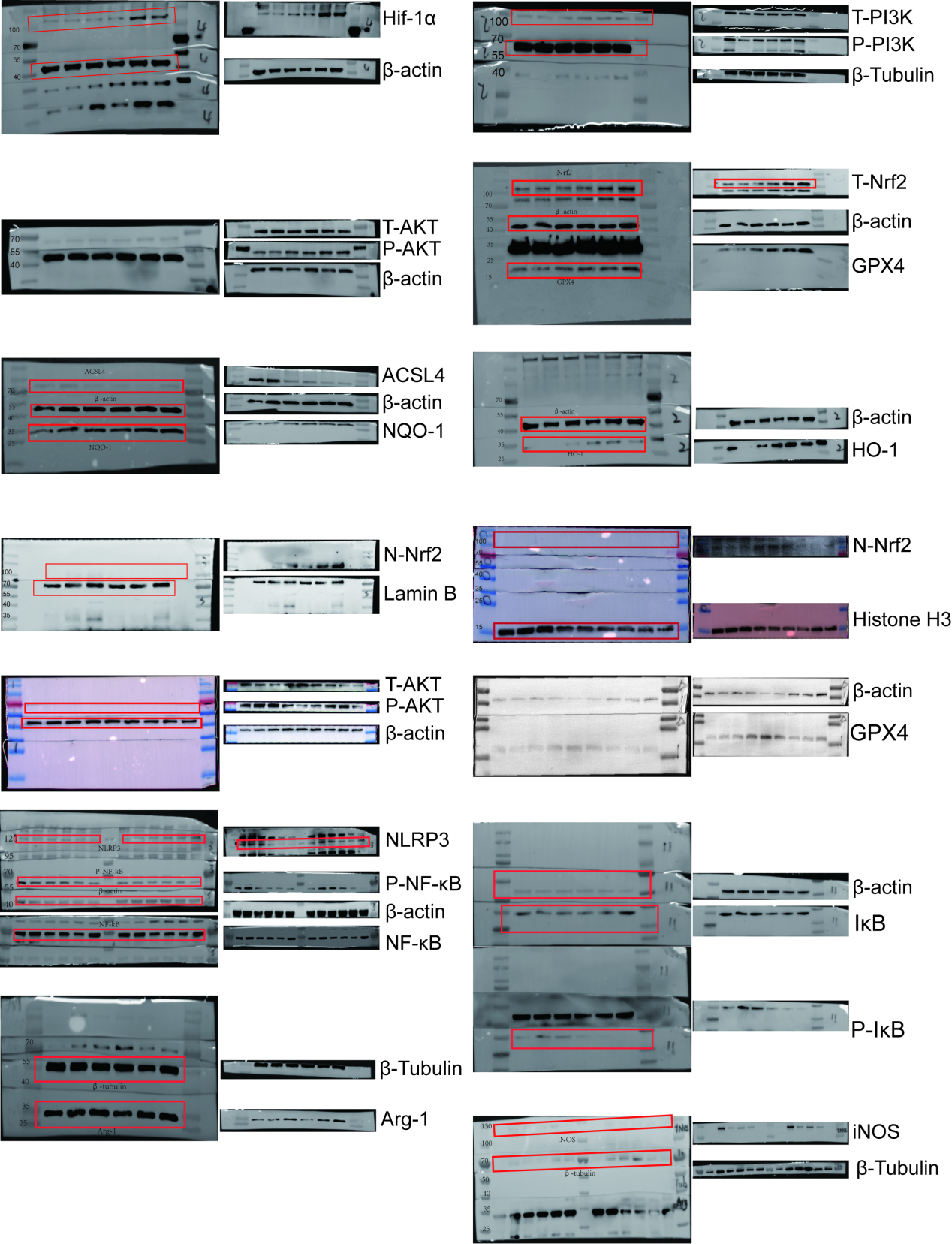


**Figure S25. Unprocessed original WB bands.** Unprocessed original WB bands in Fig.7 and Fig.8. *HIF-1* α hypoxia inducible factor 1 subunit alpha, *PI3K* phosphatidylinositol 3-kinase, *P-PI3K* phosphorylated phosphatidylinositol 3-kinase, *AKT* protein kinase b, *P-AKT* phosphorylated protein kinase b, *Nrf2* nuclear factor erythroid 2-related factor 2, *NQO1* nad(p)h quinone oxidoreductase, *HO-1* heme oxygenase 1, *GPX4* glutathione peroxidase 4, *ACSL4* acyl-coa synthetase long chain family member 4, *iNOS* inducible nitric oxide synthase, *Arg-1* arginase-1, *IL* interleukin, *TNF-α* tumor necrosis factor-alpha, *Iκk* inhibitor of nuclear factor kappa-b kinase, *IκB* inhibitor of nuclear factor kappa-b, *NF-κB* nuclear factor kappa-b, *NLRP3* nod-like receptor family pyrin domain containing 3

**Table S1. Random blood glucose levels in STZ-induced diabetic mice at baseline, 1 week, and 2 weeks post-modeling**

| Lable | Random blood glucose(mM) | | | Lable | Random blood glucose(mM) | | |
| --- | --- | --- | --- | --- | --- | --- | --- |
|  | Normal | 1week | 2week |  | Normal | 1week | 2week |
| 111 | 6.8 | 16.5 | 18.2 | 171 | 5.2 | 15.1 | 19.8 |
| 112 | 7.3 | 15.8 | 21.5 | 177 | 8.1 | 15.6 | 20.3 |
| 113 | 5.9 | 17.2 | 18.9 | 178 | 4.9 | 18.9 | 17.6 |
| 115 | 8.4 | 19.7 | 19.1 | C8 | 6.7 | 16.3 | 18.4 |
| 117 | 5.1 | 19.1 | 23.8 | C9 | 7.8 | 13.8 | 20.1 |
| 118 | 9.2 | 16.2 | 21 | C16 | 4.5 | 20.8 | 26.9 |
| 120 | 6.3 | 16.6 | 18.5 | C19 | 7.1 | 19.5 | 19.3 |
| 161 | 7.9 | 18.9 | 20.5 | C20 | 5.6 | 19.3 | 17.2 |
| 162 | 8.8 | 17.8 | 19.7 | C21 | 6.2 | 21.1 | 18.7 |
| 163 | 5.4 | 19.9 | 27.1 | C23 | 8.9 | 18.1 | 20.8 |
| 164 | 7.5 | 18.5 | 19.5 | C91 | 4.8 | 20.5 | 16.8 |
| 165 | 6.1 | 22.7 | 28.1 | C92 | 7.6 | 18.3 | 20 |
| 166 | 9 | 17.9 | 21.2 | C94 | 5.3 | 21.9 | 27.9 |
| 168 | 4.7 | 16.3 | 19 | C97 | 8.3 | 13.1 | 19 |
| 169 | 7.2 | 18 | 20.2 | C99 | 6.9 | 19.6 | 18.6 |
